# *FOXO3* longevity interactome on chromosome 6

**DOI:** 10.1111/acel.12625

**Published:** 2017-07-19

**Authors:** Timothy A. Donlon, Brian J. Morris, Randi Chen, Kamal H. Masaki, Richard C. Allsopp, D. Craig Willcox, Ayako Elliott, Bradley J. Willcox

**Affiliations:** ^1^ Department of Research Genetics Laboratory Honolulu Heart Program/Honolulu‐Asia Aging Study (HAAS) Kuakini Medical Center Honolulu Hawaii; ^2^ John A. Burns School of Medicine University of Hawaii Manoa Honolulu Hawaii; ^3^ Basic & Clinical Genomics Laboratory School of Medical Sciences and Bosch Institute University of Sydney Sydney NSW Australia; ^4^ Department of Geriatric Medicine John A. Burns School of Medicine University of Hawaii Honolulu Hawaii; ^5^ Department of Human Welfare Okinawa International University Okinawa Japan

**Keywords:** fluorescence *in situ* hybridization, *FOXO3*, gene–gene interactions, single nucleotide polymorphisms, transcription factor binding

## Abstract

*FOXO3* has been implicated in longevity in multiple populations. By DNA sequencing in long‐lived individuals, we identified all single nucleotide polymorphisms (SNPs) in *FOXO3* and showed 41 were associated with longevity. Thirteen of these had predicted alterations in transcription factor binding sites. Those SNPs appeared to be in physical contact, via RNA polymerase II binding chromatin looping, with sites in the *FOXO3* promoter, and likely function together as a *cis*‐regulatory unit. The SNPs exhibited a high degree of LD in the Asian population, in which they define a specific longevity haplotype that is relatively common. The haplotype was less frequent in whites and virtually nonexistent in Africans. We identified distant contact points between *FOXO3* and 46 neighboring genes, through long‐range physical contacts via CCCTC‐binding factor zinc finger protein (CTCF) binding sites, over a 7.3 Mb distance on chromosome 6q21. When activated by cellular stress, we visualized movement of *FOXO3* toward neighboring genes. *FOXO3* resides at the center of this early‐replicating and highly conserved syntenic region of chromosome 6. Thus, in addition to its role as a transcription factor regulating gene expression genomewide, *FOXO3* may function at the genomic level to help regulate neighboring genes by virtue of its central location in chromatin conformation via topologically associated domains. We believe that the *FOXO3* ‘interactome’ on chromosome 6 is a chromatin domain that defines an aging hub. A more thorough understanding of the functions of these neighboring genes may help elucidate the mechanisms through which *FOXO3* variants promote longevity and healthy aging.

## Introduction

Forkhead/winged helix box gene, group O (FoxO) proteins are a set of evolutionarily conserved transcription factors involved in sensing and maintaining energy homeostasis. At the cellular level, they play roles in regulation of gluconeogenesis and glycogenolysis by insulin signaling, protection against environmental and biological stressors, cell proliferation, differentiation, survival, cell cycle arrest, apoptosis, DNA repair, inhibition and promotion of differentiation, immune cell regulation, carcinogenesis, and stem cell quiescence/maintenance [see reviews: (Morris *et al*., 2015; Martins *et al*., 2016)]. They function at both the protein–protein level and at the DNA–protein level, where they bind with other elements to promote cell‐specific gene regulation. At the whole body level, FoxOs regulate a wide array of processes that include cell growth and proliferation, tumor suppression and cell cycle regulation (via Smad‐dependent transforming growth factor β), muscle homeostasis (via phosphoinositide‐3 kinase), and regulation of carbohydrate and lipid metabolism [see review: (van der Vos & Coffer, 2008)].

We first reported an association of several anonymous single nucleotide polymorphisms (SNPs) with living to at least 95 years of age (Willcox *et al*., 2008). This finding has been validated in other populations (Anselmi *et al*., 2009; Flachsbart *et al*., 2009; Li *et al*., 2009; Chung *et al*., 2010; Zeng *et al*., 2010; Bao *et al*., 2014; Soerensen *et al*., 2015). A major reason is protection against mortality from coronary heart disease (Willcox *et al*., 2016). As protein coding variants have been eliminated as a source of the genetic variability responsible for longevity (Donlon *et al*., 2012), we believe the functional variant may reside in a regulatory element where the longevity‐associated SNPs reside. Identification of a functional variant(s) in *FOXO3* will undoubtedly help to better understand the role that *FOXO3* plays in human longevity and healthy aging.

In this study, we identified 13 putative regulatory SNPs that significantly modify 18 transcription factor/enhancer binding sites that are held together in a block by linkage disequilibrium (LD) to form a *cis*‐regulatory unit that is physically connected with the *FOXO3* promoter via RNA polymerase II binding. We also find evidence that this *FOXO3*‐centered unit is connected to and central to a regulatory island on chromosome 6 that encompasses 7 268 123 bp, surrounded by gene deserts, and propose that *FOXO3*, as now known for other genes (Petkov *et al*., 2005; Zhang *et al*., 2016), is at the center of a gene network related to aging.

## Results and discussion

### Variant discovery and confirmation

**Figure 1:**
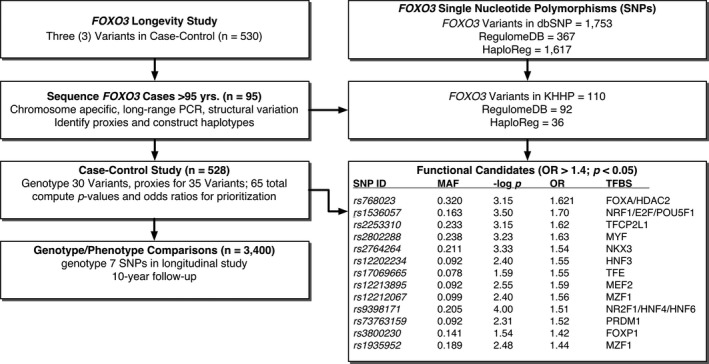
*FOXO3* functional variant identification strategy. Our original case–control study involving 530 participants identified three SNPs associated with longevity. We then sequenced DNA from 95 participants who were ≥95 years old and identified 110 SNPs that were used to screen the RegulomeDB and HaploReg databases for overlap with functional features. By limiting our search to only those SNPs that were present in our study population at a minor allele frequency (MAF) of ≥0.05, we were able to reduce the number of candidates from 1753 to 110. We used the sequencing data to identify proxies for SNPs that were not genotyped in an effort to prioritize SNPs based on significance (*P*‐values) and effect (OR). We also performed a longitudinal study to examine healthy aging phenotypes using seven SNPs – *rs2253310*,* rs2802288*,* rs9384683*,* rs2802292*,* rs2764264*,* rs3800230*, and *rs1935952*. Abbreviations: MAF, minor allele frequency; −log *P*, −log10 *P*‐value; OR, odds ratio for longevity; TFBS, transcription factor binding site.

Extensive *FOXO3* sequencing confirmed the presence of 110 variants in our subjects compared with 1753 in dbSNP (GRCh37.p13) and 199 SNPs present in the 1000 Genomes database (GRCh38.p2) with minor allele frequencies ≥0.05. We correlated the SNPs with one another in a pairwise fashion so that we could identify proxies in order to fill in information and to construct haplotypes. We initially genotyped 30 SNPs, and the proxy information identified several gaps. These were filled in by case–control analyses, so generating data on 65 SNPs, which we believe captured all of the genetic variability in the Japanese American population in Hawaii at a minor allele frequency ≥0.05. We then performed a case–control study of these 65 SNPs involving 187 cases (individuals aged ≥ 95 years) and 341 controls (individuals of average lifespan). This revealed strength (*P*‐values) and odds ratios (ORs) for association of each SNP with longevity (Table S1). Figure 1 shows the overall strategy we used for discovery, evaluation, and prioritization of the genetic variants in our study.

### Search for functional variation

While anonymous SNP associations are valuable for predicting outcomes, their real value in epidemiological analyses lies in the identification of functional variants and the pathways through which they operate. As we were able to identify all of the common genetic variants in *FOXO3,* we next sought to evaluate each SNP for its potential impact. The location of a p53 binding site in intron 2 of mouse *Foxo3* (Renault *et al*., 2011) seemed a possible candidate sequence for an effect on longevity, as p53 is a FoxO3 binding partner involved in the survival pathway (Miyaguchi *et al*., 2009). After a search of human *FOXO3* DNA, we located the corresponding p53 binding site 205‐bp 3′ of exon 2. It did not, however, overlap with any *FOXO3* variants so was dismissed. Other potential p53 binding recognition sequences in intron 2 did not exhibit the required palindromic head‐to‐head orientation for recognition. Examination of the dbSNP database revealed a possible candidate at position 108 888 423. This included two SNPs, *rs11966734* and *rs1892492*, in two half sites separated by two nucleotides, and each SNP could impact binding. These two SNPs were not, however, present in our cohort.

Screening the RegulomeDB database for all variants in *FOXO3* (chr6: 108883685–109001772; GRCh37.p13) identified 367 putative functional variants. Many of these reside in protein binding and histone modification domains found empirically (i.e., by ChIP‐Seq). They span many nucleotides, and the effect of single nucleotide changes is difficult to evaluate as canonical reference sequences are not available. We were, however, able to identify a number of transcription factor binding sites (TFBSs; DNA response elements) that allowed us to evaluate the impact of each SNP on binding affinities.

Of the 110 variants we identified by sequencing, 92 were in the RegulomeDB database predicted to significantly modify enhancers/TFBSs). These were evaluated for their potential effects by comparing the variant with the accepted TFBS canonical sequences. Similarly, we searched the HaploReg database for functional variants as well as expression‐related quantitative trait loci (eQTLs) and identified 1617 variants. Of these, 36 were present in our sequencing database and several were expected to significantly modify transcription factor binding. Many of the ‘functional’ SNPs identified in these databases technically overlap a TFBS. However, upon close examination, based on predicted differences in affinities between variants and reference nucleotides, they were not expected to significantly modify binding. We next evaluated the impact of the 92 SNPs in a subset of our case–control study and, based on *P*‐values and ORs for longevity, we were able to eliminate all but 13 SNPs (Fig. 1 and Table S1).

**Figure 2:**
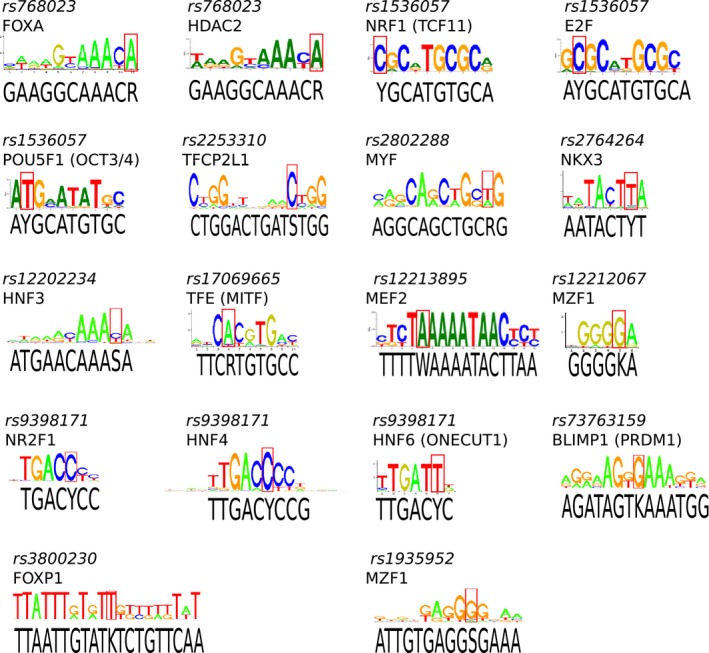
Recognition logos for transcription factor binding sites in *FOXO3*. There were 13 SNPs that modify 18 transcription factor binding sites (i.e., canonical sequences). Their logos (colored) are shown, with the height of the nucleotide designating the strength of requirement for efficient binding. The genomic sequence (black) is shown below the logo. The SNP *rs768023* modifies both FOXA and HDAC2 sites where the major allele is an A and minor allele a G, which is predicted to eliminate binding at both sites. The SNP *rs1536057* modifies NRF1, E2F, and POU5F1 binding sites, in which the major (C) allele is replaced by a T. The minor allele is predicted to abolish NRF1 and E2F binding but create a POU5F1 site. The minor allele (C) of *rs2253310* would create a TFCP2L1 site. The minor allele (G) of *rs12212067* would create an MZF1 site. The minor allele (C) of *rs1935952* would abolish an MZF1 site. The minor allele (T) of *rs1536057* would abolish NRF1 and E2F sites and create a POU5F1 site. The minor allele (C) of *rs2764264* would abolish an NKX3 site. The minor allele (C) of *rs2253310* would create a TFCP2L1 site. The minor allele (G) of *rs17069665* would abolish a TFE site. The minor allele (G) of *rs3800230* would abolish a FOXP1 site. The minor allele (C) of *rs9398171* would create NR2F1 and HNF4 sites, but abolish an HNF6 site. The minor allele (G) of *rs12202234* would abolish an HNF3 site. The canonical recognition motifs are shown in color, where the height of the letter indicates the strength of the requirement for that nucleotide. The red box denotes the location of the SNP in the recognition site, and the sequence below the motif is the genomic *FOXO3* sequence. R = ‘A’ or ‘G’; Y = ‘C’ or ‘T’; S = ‘G’ or ‘C’; W = ‘A’ or ‘T’; and K = ‘T’ or ‘G’.

The 13 putative functional variants are predicted to modify the binding affinities of 18 transcription factors. Figure 2 shows the canonical sequences for all 18 of the putative regulatory transcription factors. In the case of SNP *rs2253310* (C>G transversion), the minor allele (*G*; which is associated with longevity) should impact binding of TFCP2L1. TFCP2L1 (CRTR1) is a ubiquitously expressed member of the CP2 transcription factor family that is a transcriptional suppressor involved in stem cell replenishment and self‐renewal (Chen *et al*., 2008; Ye *et al*., 2013; Takashima *et al*., 2014). The minor allele would be expected to increase *FOXO3* expression in cell types that express TFCP2L1 (breast, cervix, colon, esophagus, kidney, salivary gland, prostate, skin, stomach, thyroid, and vagina). In the case of SNP *rs2802288* (G>A transition), the minor allele (*A*) could create a binding site for the transcription factor MYF. In the case of *rs9398171*, the minor allele (*C*) would create binding sites for two transcription factors, NR2F1 and HNF4, both of which are involved in intermediary metabolism, including glucose, fatty acid, cholesterol, and xenobiotic and drug metabolism (Hwang‐Verslues & Sladek, 2010). Ten other SNPs also appear to significantly overlap TFBSs. The functions of these transcription factors (summarized in Table S2) involve common biological pathways, including muscle homeostasis, growth/differentiation and stem cell maintenance, hematopoietic system maintenance, and glucose/fatty acid metabolism (energy sensing). These pathways fit well with the accepted roles that FoxOs play in model organisms. Table S3 summarizes the expression patterns for these transcription factors and show that over half of them are co‐expressed in the same tissues as *FOXO3*.

*FOXO3* SNPs exhibit a high degree of LD, particularly in intron 2 in the Japanese population. While *FOXO3* has been validated as a *bona fide* longevity gene in other cohorts, the level of significance obtained was lower than we observed in our Japanese American cohort. It is likely that LD plays a major role and that there are multiple loci for which selection occurs over a relatively long distance. To address these issues, we evaluated the LD of our 64 SNPs in three different populations that are available through the International HapMap project (https://www.ncbi.nlm.nih.gov/variation/tools/1000genomes/) (International HapMap *et al*., 2010). Based on SNPs with minor allele frequencies ≥0.05, we found that the majority of *FOXO3* was contained in a single haplotype block comprised of four haplotypes spanning a 122‐kb region, and six haplotypes if those with lower frequencies (<0.05) are included. Figure S1(a) illustrates the degree of LD for 37 of these 64 SNPs, the sizes of blocks, and nine of the 13 putative functional SNPs (boxed in upper panel) we identified above. The haplotypes indicate that eight of the nine variants (boxed in lower panel) are contained on a single haplotype block having a haplotype frequency of 0.083 (Fig. S1a). Four of the 13 SNPs are not shown here but are located in this region of LD (block 1). In the Caucasian population, these same SNPs are spread over three haplotype blocks, with a haplotype frequency of 0.0034 (Fig. S1b), while in the African population (YRI), the longevity haplotype is virtually nonexistent (Fig. S1c). Inclusion of all nine SNPs resulted in haplotype frequencies of 0.0678 and 0.00238 for the Asian and Caucasian populations, respectively.

### eQTLs and *cis*‐regulatory elements

Expression quantitative trait loci (eQTLs) are genomic loci that contribute to variation in expression levels of mRNAs. Our 13 SNPs should be considered as excellent candidates for eQTLs. They may, however, function only in specific tissues and under specific physiological conditions, such as biological stress. The GTEx database (http://gtexportal.org/home/) found 32 SNPs that were associated with *FOXO3* expression in transformed fibroblasts. However, all were located in the neighboring gene, *LACE1* **.** The HaploReg database (http://www.broadinstitute.org/mammals/haploreg/haploreg.php) revealed an enormous number of *FOXO3* eQTLs because of the high degree of LD in *FOXO3*. The FuncPred database (http://manticore.niehs.nih.gov/snpinfo/snpfunc.htm) indicated 82 SNPs that were predicted to be functional. GRASP could not be used as it requires phenotype data. At this time, it is not possible to discern what role, if any, these individual 13 SNPs have on quantitative differences in gene expression because of their high degree of LD. The minor allele of SNP *rs2802292*, which is in LD with the 13 SNPs we describe, is associated with elevation in *FOXO3* expression in skeletal muscle (Banasik *et al*., 2011).

### *FOXO3* SNPs relative to chromatin contacts and *cis*‐regulatory sites

The 13 putative functional SNPs in *FOXO3* were mapped relative to known TFBSs and RNA polymerase II (RNAPII) binding sites (Fig. S2a) using the WashU Genome Browser (Zhou *et al*., 2011). Shown are TFBSs from the Transfac database (green), noting the positions of the 13 SNPs (vertical black arrows), RNAPII chromatin immunoprecipitation sequencing (ChIP‐Seq) data (blue vertical lines), and chromatin contact points for RNAPII using chromatin interaction analysis by paired‐end tag sequencing (ChIA‐PET) (pink loops). Figure S2(a) shows there is a high density of enhancer elements in the *FOXO3* promoter and that many of these are physically linked to elements located in introns 2 and 3 by RNAPII binding sites. Taken together with the LD data in Fig. S1, this collectively indicates that the combination of these SNPs forms a *cis*‐regulatory unit (haplotype). This longevity haplotype is defined by the SNPS *rs768023* (*G*), *rs2253310* (*C*), *rs2802288* (*A*), *rs12202234* (*G*), *rs17069665* (*G*), *rs12212067* (*G*), *rs9398171* (*C*), *rs3800230* (*G*), and *rs1935952* (*C*), as shown in Fig. S1, and is more common in the Asian than Caucasian and African populations.

### Long‐range 3D chromatin organization in *FOXO3*

We were interested to see whether *FOXO3* is connected to any distant regulatory networks by chromatin contacts. Knowledge of these chromatin contact points in *FOXO3* could identify co‐regulated neighboring genes that also play roles in healthy aging and could help to expand our understanding of associated biological pathways.

To this end, we used the public databases Epigenome Browser (Zhou *et al*., 2011) and Juicebox (Rao *et al*., 2014) to search for contact points in the near neighborhood (500 kb) of *FOXO3*. We found that the *FOXO3* promoter is connected to genes *SESN1, CEP57L1, SEC63,* and *OSTM1* through CCCTC‐binding factor zinc finger protein (CTCF) contacts (Fig. S2b). CTCF binds to tens of thousands of sites across the genome using different combinations of its 11 zinc finger domains to bind different DNA target sequences and proteins. Sestrin‐1 (encoded by *SESN1*) responds to amino acid stimulation, regulates the response to reactive oxygen species, and is a negative regulator of mechanistic target of rapamycin complex 1 (TORC1) signaling. The TORC1 complex functions as a nutrient/energy/redox sensor and controls protein synthesis. Centrosomal protein CEP57L1 is involved in microtubule dynamics, sister chromatid segregation, and cell cycle arrest. Both SEC63 and OSTM1 are involved in protein degradation. SEC63 is a chaperon that translocates across the endoplasmic reticulum and may be involved in the unfolded protein response, an important step in the response to cellular damage. Mutations in *SEC63* have been reported in patients with polycystic kidney and liver disease. OSTM1 is involved in the degradation of G proteins via the ubiquitin‐dependent proteasome pathway. OSTM1 binds to members of subfamily A of the regulator of G‐protein signaling (RGS) family through an *N*‐terminal leucine‐rich region. OSTM1 has E3 ubiquitin ligase activity and is highly conserved from flies to humans. Defects in *OSTM1* may cause the autosomal recessive, infantile malignant form of osteopetrosis. Collectively, these genes belong to pathways that involve nutrient sensing, response to cellular stress/damage, cell division, and autophagy (Table S4).

A search for longer range *FOXO3* contacts identified a 7 268 123‐bp island of connected, and perhaps co‐regulated, genes on chromosome 6 that are surrounded by gene deserts (Fig. S2c). These direct distal contacts included 46 genes. Table S4 summarizes the functions of those genes. The majority are involved in cell growth, development, mitosis, cell cycle checkpoint, protein degradation, and gene regulation. Genes that are co‐regulated often share other genomic qualities in addition to chromatin contacts. One example is timing of DNA replication during the S‐phase of the cell cycle. *FOXO3* is at the center of an early‐replicating domain on chromosome 6 that occupies a region ~7 Mb in length (positions 105 000 000–112 600 000) (Woodfine *et al*., 2005; Weddington *et al*., 2008). Figure S3(a) shows the timing of replication for chromosome 6q21, with *FOXO3* at the center, Fig. S3(b) shows origins of replication for the same region, and Fig. S3(c) shows that there are origins of replications in *FOXO3* introns 2 and 3. We noticed that the synteny, intergenic contacts, and replication characteristics are conserved in the mouse (data not shown). By comparing the evolutionary conservation of this region, using the online database ‘Genomicus’ (Louis *et al*., 2015), we found that the group of genes extending from *C6orf203* through *PPIL6* (Fig. S4) is conserved in Euteleostomi (which is a successful clade that includes more than 90% of living species of vertebrates, also known as ‘bony vertebrates’, and dates to a common ancestor ~420 million years before the present). We did not find any significant connections between chromosome 6q21 and other chromosomes (Kaufmann *et al*., 2015).

**Figure 3:**
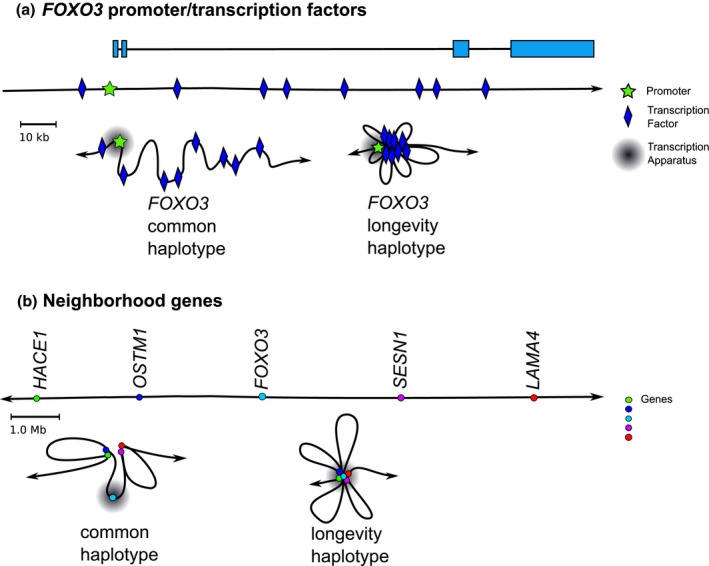
Effects of SNPs on chromatin contacts in *FOXO3* and neighborhood. (a) Aggregation of transcriptional components in *FOXO3*. Proposed mechanism whereby the pro‐longevity haplotype influences the aggregation of transcription factors (blue diamonds) with the *FOXO3* promoter (green star). The aggregation promotes *FOXO3* to migrate toward the transcriptional apparatus (gray sphere) and to promote aggregation of neighboring genes. For simplicity, only eight transcription factors/TFBSs are shown. (b) Aggregation of *FOXO3* neighboring genes. The coalescence of all the *FOXO3* transcriptional components would further integrate chromatin contacts (i.e., looping via topologically‐associated domains [TADs]) between neighboring genes on chromosome 6q21 to enable transcriptional control (transcription apparatus, gray sphere) in specific tissues. Colored circles denote *FOXO3* and, for simplicity, only four of its 46 neighboring genes on chromosome 6q21.

Figure 3 is a proposed model depicting how the longevity haplotype might influence the chromatin conformation and expression of neighboring genes on chromosome 6q21. The longevity SNPs would promote aggregation of transcription factors in introns 2 and 3 with the *FOXO3* promoter to positively influence expression. Aggregation of the *FOXO3* gene could further promote chromatin contacts with its neighboring genes to enable their tissue‐specific expression. In *Drosophila,* over 20% of the genome is composed of large domains of adjacent and similarly expressed genes, each having between ten and 30 members (Spellman & Rubin, 2002). In humans, such genomic domains are likely to be much larger, due to the larger size of the human genome.

### Experimental validation of interaction of *FOXO3* with neighboring genes

**Figure 4:**
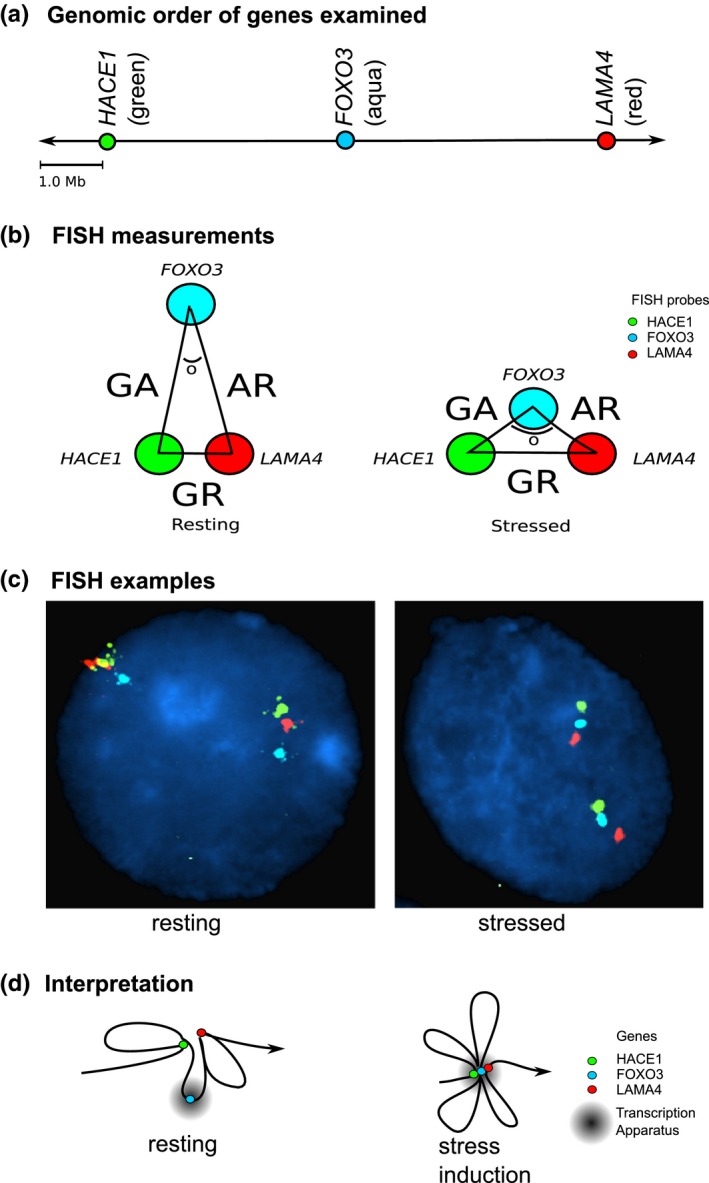
Effect of stress on *FOXO3* interaction with neighborhood genes. FISH was used to localize *FOXO3* and the two most distant genes, *HACE1* and *LAMA4*, in the *FOXO3* neigborhood in lymphoblastoid cell lines in response to cellular stress caused by 200 μm H_2_O_2_ + serum deprivation. (a) Shows the order of the three genes and color of fluorescent dye. (b) Shows the measurements taken. (c) Is an example of FISH results for one of ten lymphoblastoid cell lines from different subjects. (d) Depicts a model in which neighboring genes aggregate to form a stronger transcription complex in response to stress (only the three genes indicated, of 46 in the region, are shown).

Fluorescence *in situ* hybridization (FISH) of *FOXO3* and the neighborhood genes *HACE1* and *LAMA4*, located at extreme ends of the 7.3 Mb domain (*HACE1* being 3 573 231 bp proximal and *LAMA1* being 3 694 892 bp distal to *FOXO3*) showed that H_2_O_2_ stress‐induced activation of *FOXO3* in lymphoblastoid cell lines caused movement of *FOXO3* toward *HACE1* and *LAMA4* as they are drawn into the transcription apparatus (Fig. 4 and Fig. S5a and b). Higher *FOXO3* mRNA expression was seen in response to H_2_O_2_‐induced stress in cell lines heterozygous for the longevity‐associated *G* allele of *FOXO3* SNP *rs2802292* than in cell lines with the *TT* genotype (Fig. S5c). A significant, but less marked, increase in *HACE1* was seen in response to H_2_O_2_‐induced stress and did not differ by *FOXO3* genotype of the cells. *LAMA4* was not consistently expressed in these cell lines.

Figure 4(d) is a proposed model depicting the influence of stress‐induced *FOXO3* expression on chromatin conformation and expression of neighboring genes on chromosome 6q21. *FOXO3*, when activated by stress, moves from a ‘quiescent’ location in the cell nucleus to one that promotes expression. We propose that neighboring genes are drawn into the transcriptional domain whereby they become co‐linear and expressed at higher rates. The longevity SNPs would promote an increase in *FOXO3* expression, but only indirectly influence expression of other genes in the neighborhood. Aggregation of *FOXO3* with the longevity haplotype could further promote chromatin contacts with its neighboring genes to enable their tissue‐specific expression.

### Tissue expression patterns between *FOXO3* and neighboring genes

The majority of the syntenic loci are found to be largely co‐expressed, according to the public database ‘GeneMania’, the source of most of the data in the Gene Expression Omnibus (GEO) (Warde‐Farley *et al*., 2010). To determine tissue‐specific expression patterns, we compared *FOXO3* and its neighboring genes using the GTEx database. Table S5 summarizes the expression pattern for *FOXO3* across 53 tissues. While *FOXO3* is ubiquitously expressed, some tissues, such as adipose tissue, brain, fibroblasts, heart, and lung, show particularly high expression. Sixteen other genes had similar expression patterns and pathways relevant to healthy aging/longevity. These include genes involved in autophagy (*HACE1*,* ATG5, SOBP, SEC63*, FIG4), energy sensing (*OSTM1*), stress response (*SNX3),* nutrient sensing (*SESN1*), cell proliferation (*CD164*), apoptosis (*MICAL*1), cell proliferation (*CDC40*,* RPF2, FYN, TUBE1, GTF3C6*), and stem cell maintenance (*AMD1)*.

### Previous epidemiological studies on chromosome 6q21

Genomewide association studies (GWAS) have identified disease risk and phenotype loci in the vicinity of *FOXO3* (Table S6). The vast majority were associated with phenotypes associated with response to insulin, tissue growth, hematopoiesis, and inflammation. This list is likely an underestimate as many GWAS results are not readily indexed in the literature owing to the high cutoff for genomewide significance, so excluding associations of moderate risk. For example, the association between *FOXO3* SNP *rs2153960* and plasma IGF‐1 level (*P *=* *5.1 × 10^−7^) (Kaplan *et al*., 2011) just failed to reach genomewide significance (*P *=* *5 × 10^−8^).

### General findings of the study

We identified 13 *FOXO3* variants that significantly modify transcription factor binding. These are clustered together on a contiguous segment of chromosome 6 that forms a longevity haplotype. We found this haplotype is more frequent in the Japanese American population than white American or Yoruba African. We present evidence that the *FOXO3* promoter is physically connected with transcription factors and TFBSs in introns 2 and 3 via RNAPII binding. RNAPII sites help form promoter–promoter and promoter–regulatory interactions between genes that are actively and cooperatively transcribed (Li *et al*., 2012). It is known that the promoter, enhancer(s), and other *cis*‐regulatory elements are generally clustered into islands of co‐regulated chromatin that define a long‐range regulatory landscape. Chromatin interaction maps have revealed patterns that can best be described by two compartments, ‘A’ and ‘B’, that alternate along chromosomes and generally have a size of ~5 Mb each. ‘A’ compartments preferentially interact with other ‘A’ compartments and ‘B’ with other ‘B’. The A/B compartments correlate with indicators of transcriptional activity such as DNA accessibility, gene density, replication timing, GC content, and several histone marks, suggesting that A compartments are largely euchromatic, transcriptionally active regions [see review: (Dekker *et al*., 2013)]. Risk‐associated variants (SNPs) can modulate distant chromatin contacts and have profound effects on allele‐specific gene expression [see review: (Zhang *et al*., 2014)]. For example, the SNP *rs4784227* overlapped a FoxA1 binding site at a distal regulatory element in *TOX3*, a breast cancer‐associated gene (Cowper‐Sal lari *et al*., 2012) and alters *TOX3* expression. In *OCA2*, the SNP *rs12913832* modulates chromatin loop formation and is associated with darker melanocyte pigmentation (Jing *et al*., 2008).

Our findings provide a novel perspective on the mechanism by which *FOXO3* mediates its effect on lifespan and aging. We propose that the longevity genotype is actually a collection of 13 variants that when considered in the combination noted (i.e., as a haplotype) can lead to an appropriate activation of *FOXO3* and that *FOXO3* serves as a vehicle for the activation of neighboring genes, thus increasing the likelihood of living to extreme old age. Genetic diversity in the population is likely to be a major factor, as LD and population structure are factors that maintain this haplotype as a functional unit. Genetic epidemiological studies of risk‐associated phenotypes are often difficult to validate when the associated variants are in noncoding regions of the genome. This is because they rely on a degree of LD – the greater the degree of LD the greater the success of discovery. Cohorts that are relatively homogeneous, such as that found in the Kuakini Honolulu Heart Program cohort of Japanese Americans here, offer a unique opportunity to discover moderate‐risk associations.

Our study finds evidence that *FOXO3* is physically connected, through chromatin looping, with 46 other genes on chromosome 6 and forms a larger functional unit. Together, these span a region of 7 268 123 bp. Not surprisingly, co‐regulated genes are often localized in close proximity over many kb. *Cis*‐regulatory elements are brought together into co‐regulated islands and multiple islands are brought together into a functional neighborhood, or ‘archipelago’ by chromatin looping (see review: (Montavon *et al*., 2011). These chromatin islands (TADs) can be detected through cross‐linking experiments and are of the order of several hundred kb, while archipelago connections can be on the order of 3–5 Mb (Dixon *et al*., 2012; Crane *et al*., 2015). Larkin *et al*. (2009) propose that natural selection acts on the genome to maintain combinations of genes and their regulatory elements that are essential to fundamental processes of development and biological organization. They identified a 1 237 140‐bp region on chromosome 6 that contains *FOXO3* in a homologous syntenic block that is maintained in all amniotes. This block (having coordinates 108 502 628–109 739 768; Hg17, build 35) includes the genes *OSTM1*,* NR2E1*,* SNX3*,* LACE1*,* FOXO3*,* LINC00222*,* ARMC2*,* SESN1* and *CEP5*, supporting the premise that it is highly conserved. Also, the group of genes extending from *C6orf203* through *PPIL6* (Fig. S4) is highly conserved. This cluster of genes on chromosome 6q21 forms an early‐replicating unit of ~7 Mb in length (Fig. S3), providing further evidence that the genes belong to a functional neighborhood. There is strong evidence that the replication machinery is cohesive with transcriptional factories (Hassan *et al*., 1994).

Systems‐level analyses (Zhang *et al*., 2016)have revealed that aging genes are more likely to involve network hubs, play important roles in communication among different functional pathways, and physically interact with and be co‐expressed with genes essential to those pathways. Aging genes are characterized by high expression levels across a large number of tissue types. This points to a high level of connectivity. We believe that the *FOXO3* interactome on chromosome 6 is a chromatin domain that defines an aging hub. Genes sharing such chromatin domains are often co‐regulated in specific tissues (Nora *et al*., 2012) [see review: (Al‐Shahrour *et al*., 2010)]. There is a significantly higher degree of co‐expression of genes belonging to a given functional class (described, e.g., by GO terms) when they are packed within a functional neighborhood than when they are located elsewhere in the genome (Al‐Shahrour *et al*., 2010). The GO terms of these 46 co‐regulated genes include the following: gene regulation, membrane trafficking, autophagy, stress response, nutrient sensing, cell division, proliferation, and differentiation. We believe that, once activated, *FOXO3* cooperates with its neighbors through chromatin contacts. In turn, these 46 genes respond in their own tissue‐specific capacities by way of pathways that include response to nutrient levels, response to cellular damage, and responses related to growth vs. senescence. One should note that this list of 46 genes includes mostly protein‐coding genes. There is a large number of uncharacterized transcripts in this 7.3‐kb region whose function is unknown. It is possible that these too could influence healthy aging and longevity.

## Experimental procedures

### Subjects

The study involved leukocyte DNA from 528 American men of Japanese ancestry from the Kuakini Honolulu Heart Program, including 187 cases (aged ≥ 95 years) and 341 controls (aged ≤ 70 years). Recruitment, demographic characteristics, and DNA extraction were as described previously (Willcox *et al*., 2008; Donlon *et al*., 2012). DNA sequencing was performed on lymphoblastoid cell line DNA from 95 participants who had attained an age 95 years or older.

### DNA sequencing

DNA from lymphoblastoid cells lines from 95 participants who had achieved a minimum age of 95 years was isolated. A method of long‐range PCR (Cheng *et al*., 1994) was used to generate seven chromosome 6‐specific products of 16–18 kb in order to avoid complications from repetitive DNA and the *FOXO3* pseudogene, *FOXO3B*/*FKHRL1P1*. Primers (Table S7) were designed using GeneScript (http://www.genscript.com) and the Primer3 (Rozen & Skaletsky, 2000) component of Geneious™ (http://www.geneious.com). Amplified products were purified using Acroprep 96 Omega 10K filter plates (Pall Corp., Port Washington, NY, USA) and were sequenced by capillary electrophoresis using an ABI Prism^®^ 3100 Genetic Analyzer (Foster City, CA, USA) together with sequencing primers shown in Table S7. The sequences generated were screened for nucleotide substitutions using the Geneious™ software package (Biomatters Ltd, Auckland, New Zealand) (Kearse *et al*., 2012). Structural variation was assessed by digesting the long‐range PCR products with *Eco*RI and/or *Hin*dIII and comparing digests on a 0.7% agarose gel.

### Genotyping

Genotyping was performed by allelic discrimination assays using TaqMan^®^ (Applied Biosystems, Inc., Foster City, CA, USA) and a Life Technologies QuantStudio 12K Flex OpenArray system.

### Haplotype grouping and linkage disequilibrium

From the sequencing data, we were able to impute haplotypes in our long‐lived study population and to identify surrogates for SNPs in order to reduce the amount of genotyping. We compared minor alleles using the Cohen's kappa coefficient to determine relatedness. LD was obtained using the program HaploView.

### Variant search

All variants were identified by sequencing and were included if two or more subjects possessed the same variant. All variants were screened on the RegulomeDB site, which includes known and predicted regulatory elements in the intergenic regions, as well as regions of DNAase hypersensitivity, binding sites for transcription factors, and promoter regions that have been shown to regulate transcription. Sources of these data included public datasets from GEO, the ENCODE project, and published literature (Boyle *et al*., 2012). Unless otherwise indicated, all locations on chromosome 6 use the GRCh37.p13 genome build (http://www.gencodegenes.org/releases/19.html).

We also screened the variants using HaploReg, which is a tool for exploring annotations of the noncoding genome at variants on haplotype blocks, such as candidate regulatory SNPs at disease‐associated loci (Ward & Kellis, 2012). Using LD information from the 1000 Genomes Project, linked SNPs and small indels can be visualized along with chromatin state and protein binding annotation from the Roadmap Epigenomics and ENCODE projects, sequence conservation across mammals, the effect of SNPs on regulatory motifs, and the effect of SNPs on expression from QTL studies. We searched haploreg version 4.1 for the region chr6:108866973‐109011102 using the Nov 5, 2015 build, hg38.

### Chromosome conformation capture/neighbor analyses

We searched points of chromatin contact of *FOXO3* on chromosome 6 using the WashU Epigenome Browser public database (http://epigenomegateway.wustl.edu/browser) (Zhou *et al*., 2011) and the Juicebox program (Rao *et al*., 2014).

### Fluorescence *in situ* hybridization

This study involved lymphoblastoid cells lines from 20 offspring of our long‐lived Japanese American subjects. Cells from ten participants heterozygous for the longevity‐associated minor (*G*) allele of *FOXO3* SNPs *rs2802292* and ten homozygous for the common (*T*) allele were compared. Cultured lymphoblastoid cell lines were treated with vehicle or 200 μm H_2_O_2_ + serum deprivation overnight. Fluorescently labeled RPC11 BAC clones 460L11 (*HACE1*; 5‐fluorescein dUTP), 249L21 (*FOXO3*; Aqua), and 28L24 (*LAMA4*; 5‐TAMRA dUTP) were obtained from Empire Genomics, Inc. (Buffalo, NY). Lymphoblast cells were fixed in Carnoy's fixative, and 5 μL probe was applied and sealed with a coverslip, denatured for 2 min at 88 °C, hybridized overnight at 37 °C, washed for 2 min in 2 × SSC at room temperature for 2 min in 0.4 × SSC/0.25% Tween, and mounted in antifade containing 1.0 μg mL^−1^ DAPI. Ten nuclei from each cell line/condition/genotype (400 total) underwent imaging using an Olympus BX‐61 epifluorescence microscope equipped with a cool charged couple device camera (JAI *CV‐M4*+*/M4*+*CL*) using CytoVision software (v 7.4, Leica Biosystems Inc., Buffalo Grove, IL). Four single bandpass filters for FITC, TRITC, Aqua, and DAPI (Chroma Technology, Bellows Falls, VT) were used. All images were obtained via 12 × 0.5 μm sections (i.e., z‐stacks) and integrated into a single file. Weighted centers were determined, and distances (in pixels) and angles between probe signals were determined using ImageJ [Fiji (Schindelin *et al*., 2012)]. The average nucleus was 10.5 μm in diameter, and data were displayed so that 1.0 unit = 100 μm. The method of 2D rather than 3D FISH was utilized because of its superior degree of probe access/hybridization and overall robust methodology, leading to stronger fluorescent signals and less laborious file management. Distances were measured from *HACE1* (green) to *FOXO3* (aqua), designated ‘GA’, from *FOXO3* to *LAMA4* (red), designated ‘AR’, and from *LAMA4* to *HACE1*, designated ‘GR’ (Fig. S5a). Angles were measured from *HACE1* to *FOXO3* to *LAMA4* (Fig. S5a). As there was a small degree of variability in the exact diameter of the nuclei, and the distances between signals, we also measured the ratios of these distances.

### Gene expression

RNA was extracted from cultured lymphoblastoid cell lines treated with vehicle or 200 μm H_2_O_2_ + serum deprivation overnight, then treated with RNAse I‐free DNAse I, and made into cDNA using RNeasy (Qiagen Inc., Germantown, MD, USA). Quantitative reverse transcriptase (qRT)–PCR was performed for *FOXO3* mRNA using assays Hs04194415 and Hs04195365 for transcript variants 1 and 2, respectively, for *HACE1* using Hs00410879, and for *LAMA4* using Hs00935293. The *FOXO3* assays were specifically chosen so as not to detect the *FOXO3B* pseudogene transcript on chromosome 17. As this assay involves a single exon, DNAase I was added to the RNA prior to cDNA synthesis. Transcript levels were quantified using the relative quantification method (minus delta delta *C*_t_) on a QuantStudio 12K Flex system.

## Conflict of interests

None declared.

## Author contributions

T.A.D., B.J.W., and K.H.M. designed the research; T.A.D., B.J.W., R.C., K.H.M., and A.E. performed the research; T.A.D. contributed new reagents/analytic tools; R.C. analyzed data; and T.A.D., B.J.M., B.J.W., K.H.M., and D.C.W. wrote the manuscript.

## Funding

This work was supported by contract N01‐HC‐05102 from the National Heart, Lung, and Blood Institute and contract N01‐AG‐4‐2149 and Grants 5 U01 AG019349‐05 and R01 AG027060‐01 (Hawaii Lifespan Study) from the National Institute on Aging. Authors BJW and TAD declare that they currently hold a US patent (number 20130295566) entitled ‘Method of Using FOXO3A Polymorphisms and Haplotypes to Predict and Promote Healthy Aging and Longevity’.

## Supporting information


**Fig. S1.** Linkage disequilibrium and haplotypes for three populations.
**Fig. S2.** Interactions between potentially functional *FOXO3* SNPS and nearby genes on chromosome 6q21.
**Fig. S3.** DNA replication of chromosome 6q21.
**Fig. S4.** Phylogeny of human chromosome 6q21.
**Fig. S5.** FISH experiments to determine the effect of H_2_O_2_ stress‐induced activation of *FOXO3* in lymphoblast cell lines on interaction with two genes (*HACE1* and *LAMA4*) flanking *FOXO3* on chromosome 6q21.

**Table S1.** *FOXO3* SNPs evaluated in this study.
**Table S2.** Transcription factors modified by variants.
**Table S3.** Expression patterns of longevity‐associated transcription factors.
**Table S4.** Genes on chromosome 6q21.
**Table S5.** Expression patterns of genes on chromosome 6q21.
**Table S6.** Published GWAS results for chromosome 6q21.
**Table S7.** Amplification and sequencing primers.
