# Supplementary material for: FOXO3 longevity interactome on chromosome 6
Source: Aging Cell. 2017 Jul 19;16(5):1016–25. doi: 10.1111/acel.12625 (PMC5595686; doi:10.1111/acel.12625)
Supplement: Supplementary file 1 — Fig. S1. Linkage disequilibrium and haplotypes for three populations. Fig. S2. Interactions between potentially functional FOXO3 SNPS and nearby genes on chromosome 6q21. Fig. S3. DNA replication of chromosome 6q21. Fig. S4. Phylogeny of human chromosome 6q21. Fig. S5. FISH experiments to determine the effect of H2O2 stress‐induced activation of FOXO3 in lymphoblast cell lines on interaction with two genes (HACE1 and LAMA4) flanking FOXO3 on chromosome 6q21. [file ACEL-16-1016-s001.pdf]

Suppl. Fig. S1. Linkage disequilibrium and haplotypes for three populations. (a) Asian (CHB + JPT)

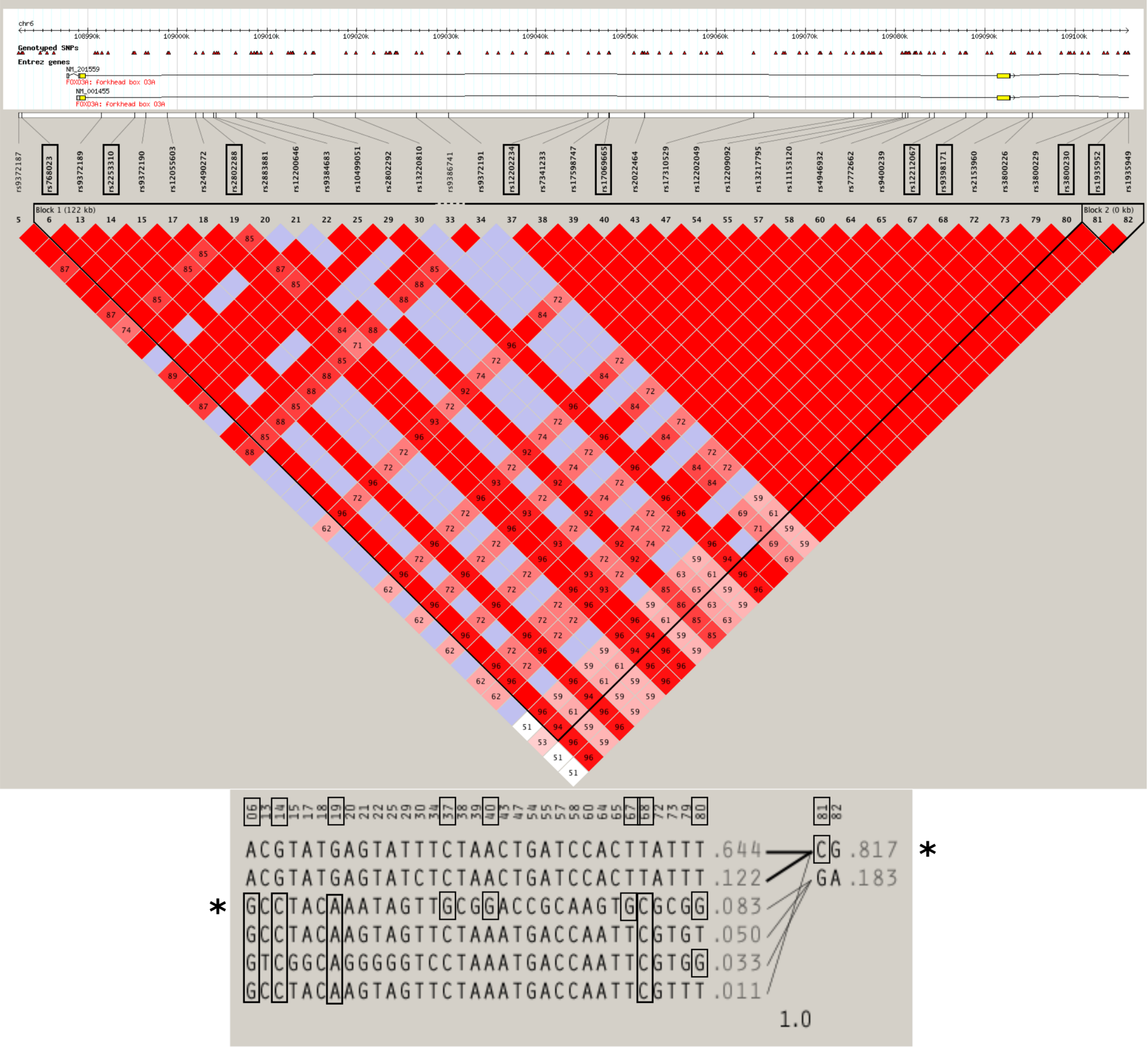

(b) Caucasian (CEU)

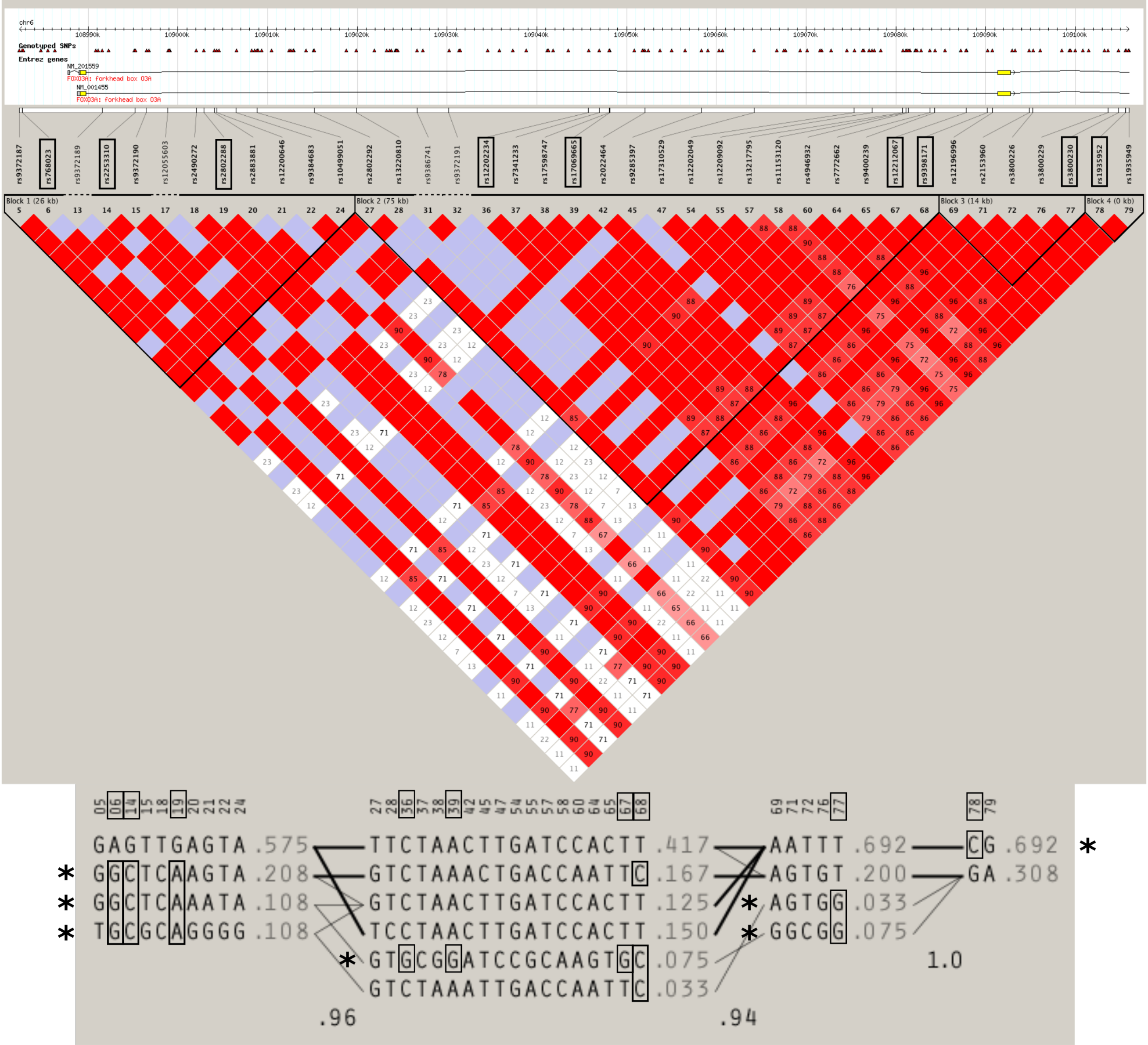

(c) African (YRI)

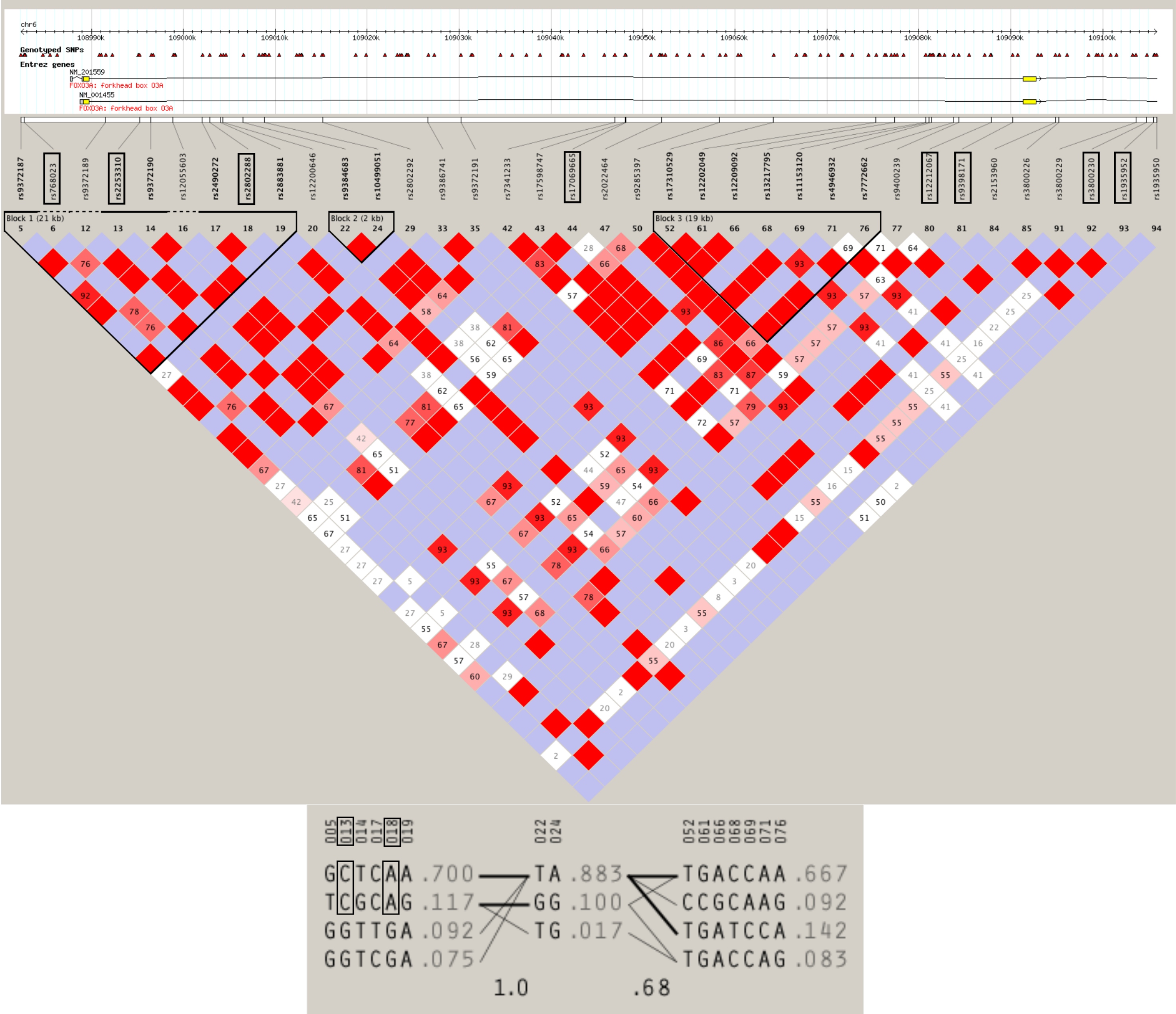

LD plot showing the 64 *FOXO3* SNPs and proxies identified using the program Haploview. Those SNPs with putative functional variation are shown in boxes (most signficant). The HapMap data include nine of the 13 putative regulatory SNPs. Each SNP is assigned a "marker number" below. The corresponding haplotypes are shown below the LD heat plot. The longevity haplotype is defined by SNPs *rs768023* (G, marker "06"), *rs2253310* (C, marker "14"), *rs2802288* (A, marker "19"), *rs12202234* (G, marker "37"), *rs17069665* (G, marker "40"), *rs12212067* (G, marker "67"), *rs9398171* (C, marker "68"), *rs3800230* (G, marker "80"), and *rs1935952* (C, marker "81"). In the Asian population, shown in part **(a)** of Fig. S1, the haplotype frequency is 0.0678 (0.083 x 0.817). In the Caucasian population, shown in part **(b)** of Fig. S1, the haplotype frequency is 0.00238 ((0.208 + 0.108 + 0.108) x 0.075 x (0.033 + 0.075) x 0.692) (HapMap data CHB + JPT release 27 phase II+III, Feb 2009 on NCBI B36 assembly, dbSNP b126. CEU release 27 phase II+III, Feb 2009 on NCBI B36 assembly, dbSNP b126). Haplotype frequency in the Caucasian population was lower: 0.00238 ((0.424 (0.208 + 0.108 + 0.108) x 0.075 x 0.108 (0.033 + 0.075) x 0.692). In the African population, shown in part **(c)** of Fig. S1 (YRI, release 27 phase II+III, Feb 2009 on NCBI B36 assembly, dbSNP b126), the nine SNPs would not likely be found in any appreciable frequency, meaning haplotype frequency would be too low to warrant consideration. Shown is the region spanning nucleotides 108,980,000–109,120,000 (from HapMap data release 27, phase II + III, Feb 2009, on NCBI B36 assembly, dbSNP b126). Red squares denote blocks that have a Hedrick's multiallelic D' = 1, whereas pink or white denote blocks that have a D' value < 1 (Hedrick 1987).

Suppl. Fig. S2. Interactions between potentially functional *FOXO3* SNPS and nearby genes on chromosome 6q21.

(a) Putative functional SNPs in *FOXO3*, cis-regulatory elements, RNAPII Chip-Seq data), and chromatin contact points for RNAPII.

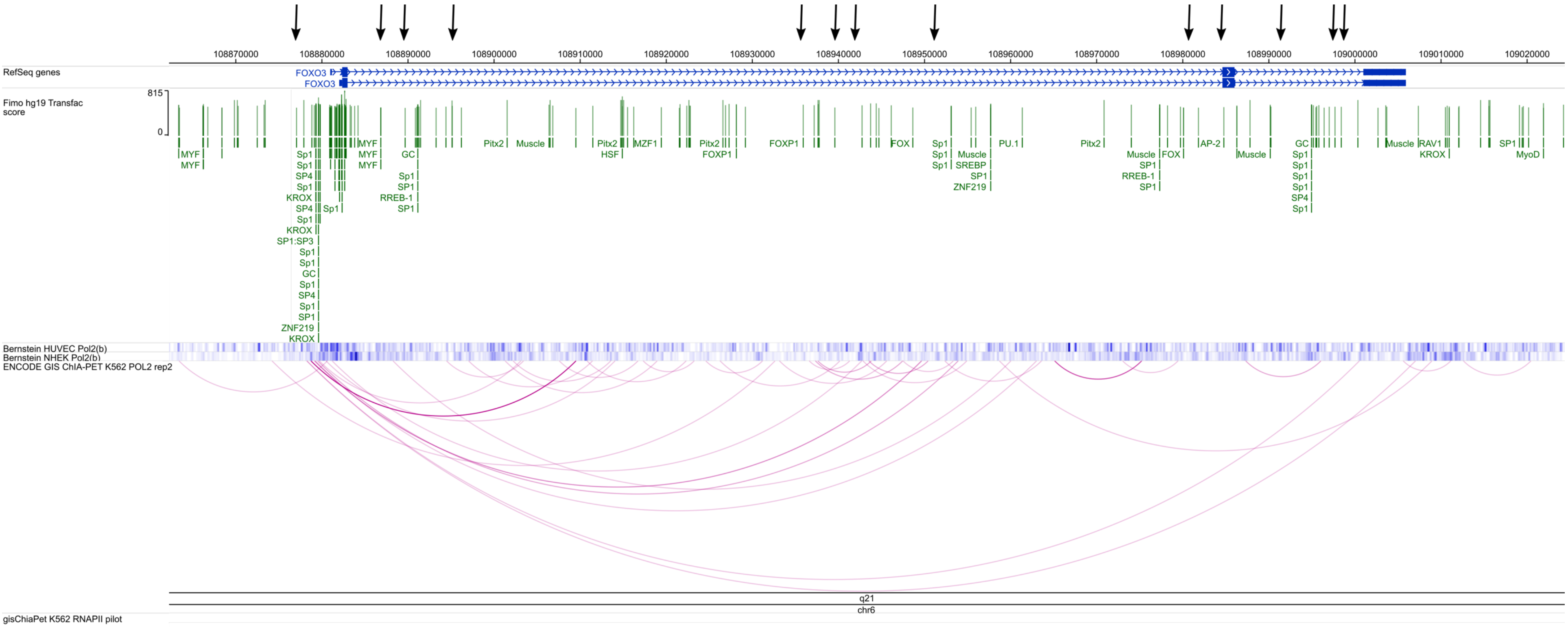

In part **(a)** of Fig. S2 the 13 putative functional SNPs of *FOXO3* were mapped relative to known transcription factor binding sites (TFBSs) and RNA polymerase II (RNAPII) binding sites (blue) using the WashU Genome Browser (Zhou et al. 2011). The TFBSs are from the Transfac database (green), noting the positions of the 13 SNPs (small vertical black arrows), RNAPII chromatin immunoprecipitation sequencing (ChIP-Seq) data (blue vertical lines), and chromatin contact points for RNAPII using chromatin interaction analysis by paired-end tag sequencing (ChIA-PET) (pink loops). This figure shows that there is a high density of enhancer elements in the *FOXO3* promoter and that many of these are physically linked to elements located in introns 2 and 3 by RNAPII binding sites. An overlay of *FOXO3* is shown (blue arrows). Data were derived from the WashU Genome Browser (Zhou et al. 2011). The RNAPII binding site data were from channels: “Berstein HUVEC Pol2(b)” and "Berstein NHEK Pol2(b)”. The RNAPII looping data are from channel “ENCODE GIS ChIA-PET K562 POL@ rep2”.

Suppl. Fig. S2 continued

(b) Short-range CTCF chromatin contacts between the proximal promoter region of *FOXO3* and neighboring genes.

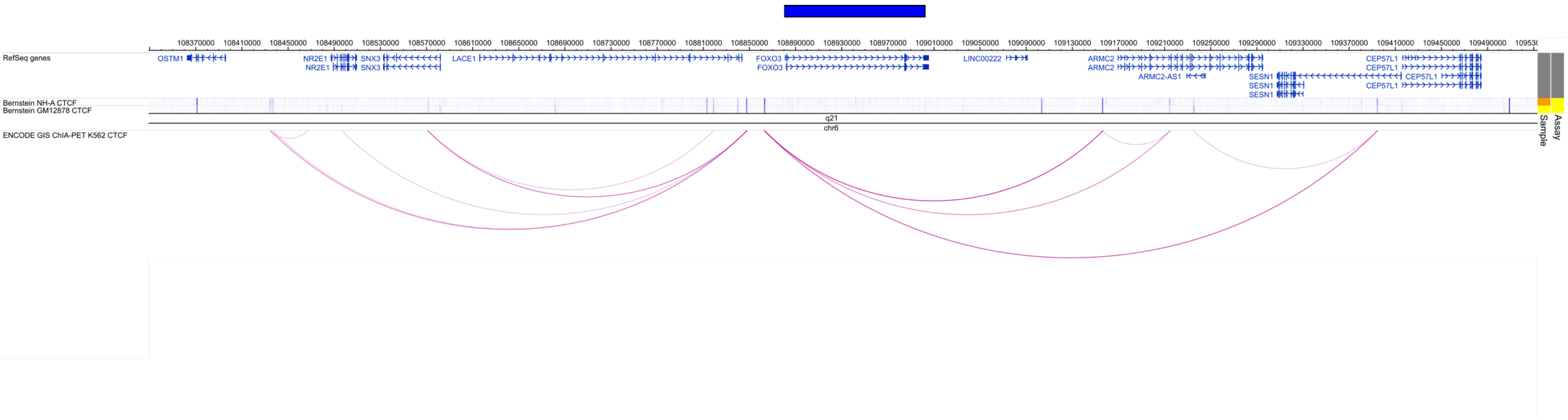

In part **(b)** of Fig. S2 short-range CTCF chromatin contacts (shown as loops) between the proximal promoter region of *FOXO3* (blue bar) and neighboring genes. The promoter region of *FOXO3* was found to connect through chromatin contacts with several neighboring genes, including *OSTM1*, *SNX3*, *SESN1* and *CEP57L1*. Shown are CTCF binding sites (blue boxes) obtained by ChiP-Seq connected through CTCF-bound chromatin looping (pink loops) obtained by ChiA-PET. Data for CTCF ChIP-Seq are from channels "Bernstein NH-A CTCF" and Berstein GM12878 CTCF". Data from ChiA-PET are from the channel "ENCODE GIS ChiA-PET K562 CTCF

Suppl. Fig. S2 continued

(c) Long-range CTCF chromatin contacts between promoter of *FOXO3* and neighboring genes.

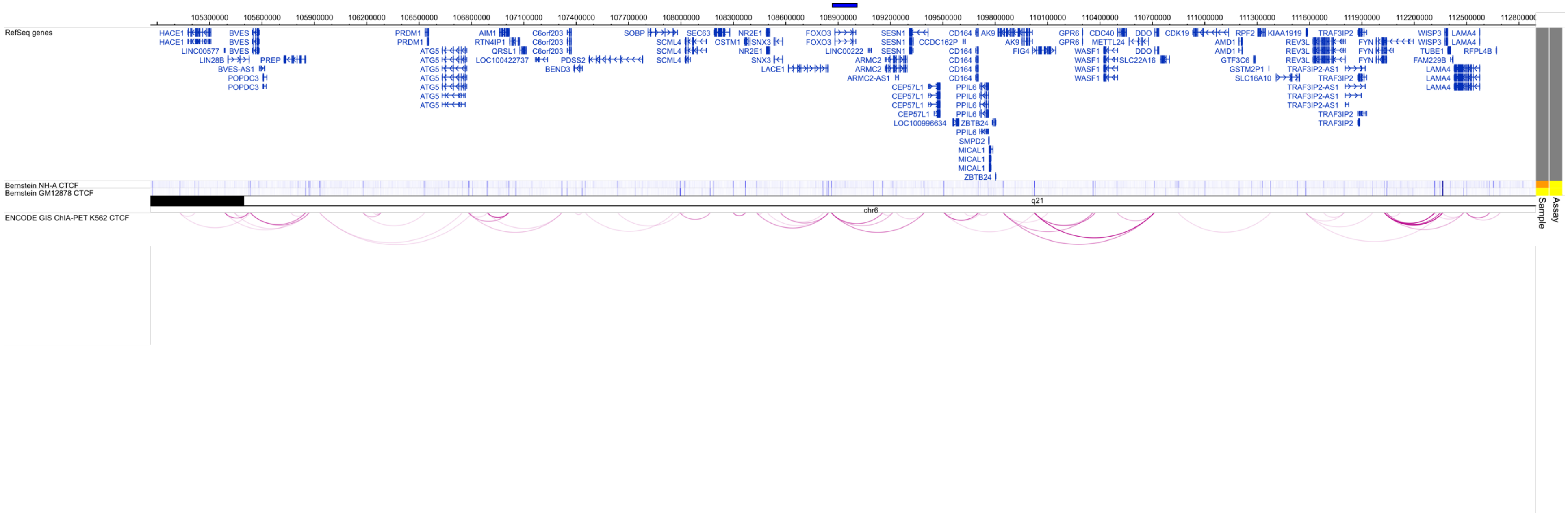

In part **(c)** of Fig. S2 long-range CTCF chromatin contacts (loops) are shown between promoter of *FOXO3* (blue bar) and neighboring genes. *FOXO3* is at the center of a 7,268,123 bp long segment on chromosome 6 that is connected through CTCF contacts. The same data files as in “(b)” are shown, except that the view has been zoomed out.

Suppl. Fig. 3. DNA replication of chromosome 6q21

(a) Timing of replication

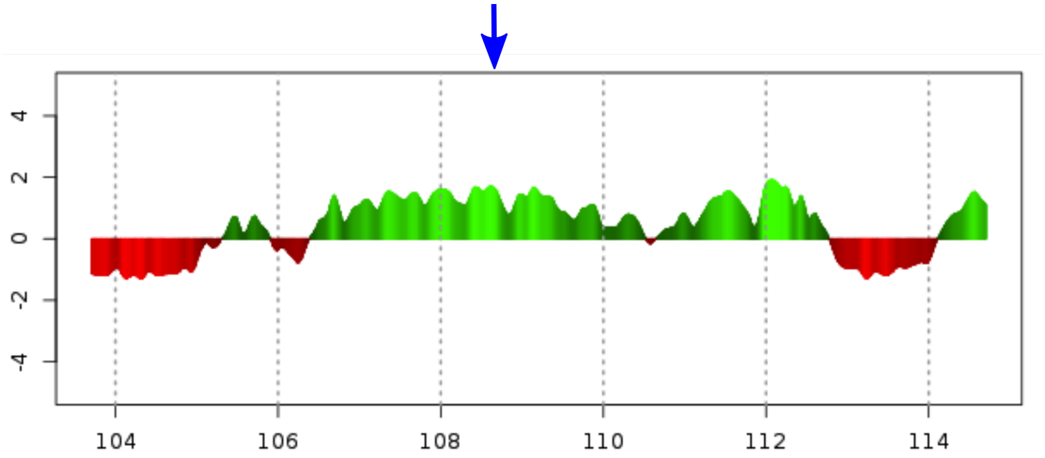

(b) Origins of replication

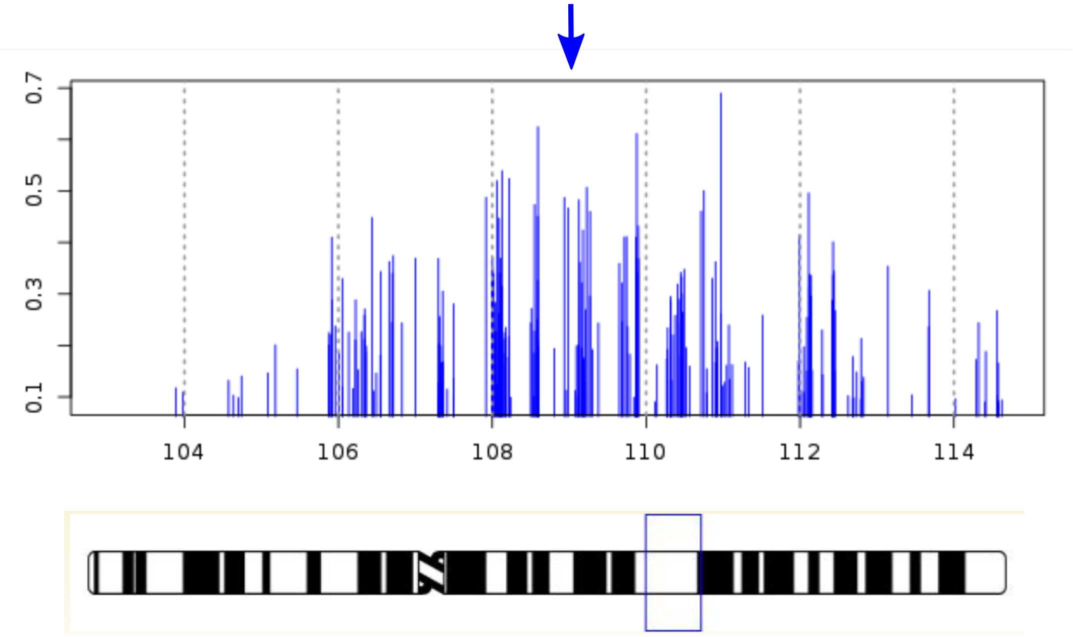

(c) Origins of replication in *FOXO3*

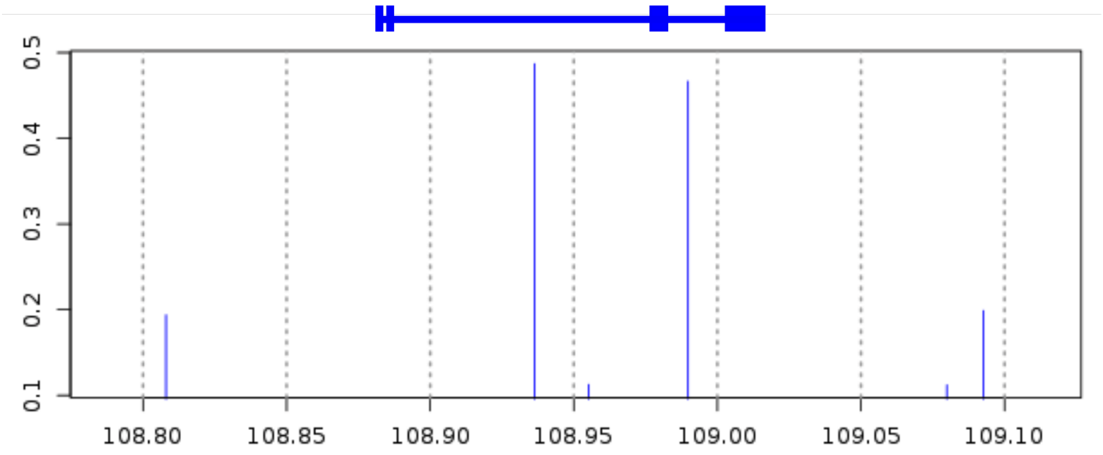

In part **(a)** of Suppl. Fig. S3 the region from 107,000,000 to 113,000,000 on chromosome 6 is early-replicating (green peaks). *FOXO3* is denoted by the blue arrow.
In part **(b)** the origins of DNA replication are shown by blue bars and appear to peak around *FOXO3* (blue arrow).
In part **(c)** there appear to be origins of DNA replication in *FOXO3* introns 2 and 3. The blue rectangles denote exons 1–4, while the blue line denotes introns.

Suppl. Fig. S4. Phylogeny of human chromosome 6q21

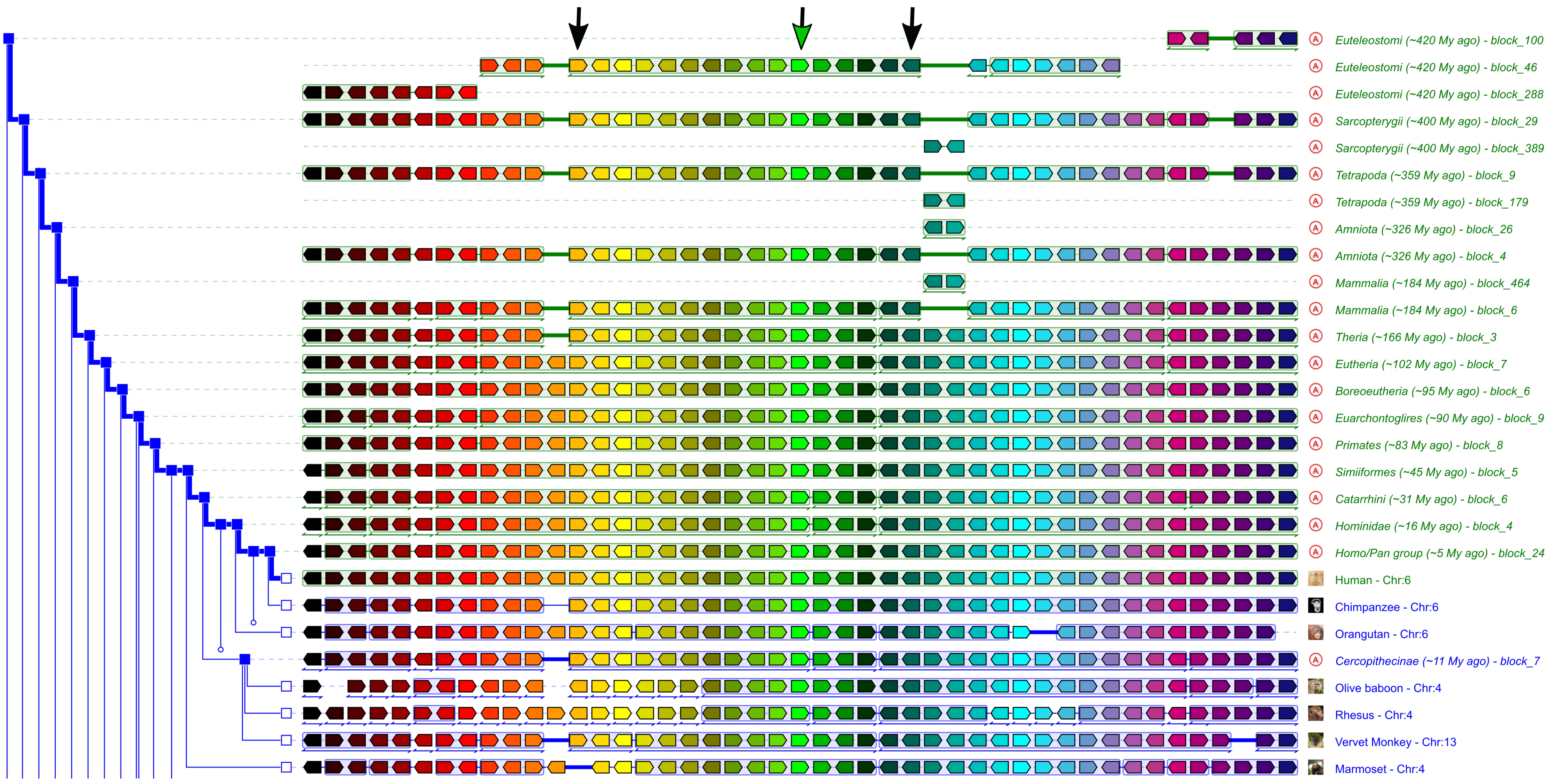

This Figure shows results of use of the public database “Genomicus” to search for conservation of the region surrounding *FOXO3*. These data are derived from Ensembl release 76, which contains genome annotations for 68 extant species. The primate order is shown in the last 13 rows, predicting a syntenic region that is conserved in the Euteleostomi clade (row 2), which dates back 420 million years. Those conserved genes shown in row 2 include the region spanning the genes *AIM1* through *METTL24*, that contain several gaps, and the region from *C6orf203* through *PPIL6* (black arrows) that has no gaps. *FOXO3* is shown by the green arrow and the human chromosome is the bottom green row labeled “human – Chr:6”.

Suppl. Fig. S5. FISH experiments to determine the effect of H_2O_2 stress-induced activation of *FOXO3* in lymphoblast cell lines on interaction with two genes (*HACE1* and *LAMA4*) flanking *FOXO3* on chromosome 6q21.

(a) Explanation of interphase chromosome measurements made.

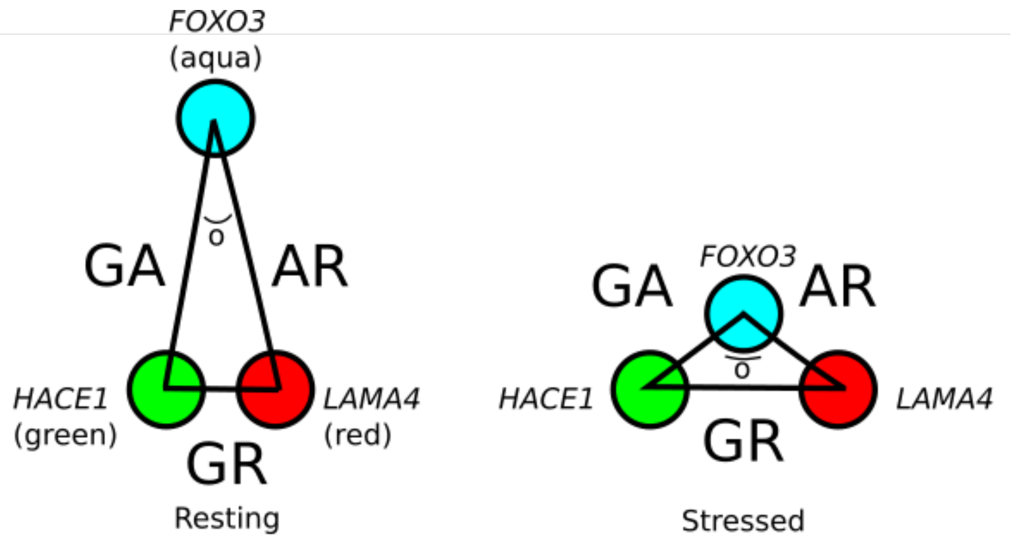

(b) Interphase chromatin organization

(i) Distances between genes comparing genotype vs. treatment

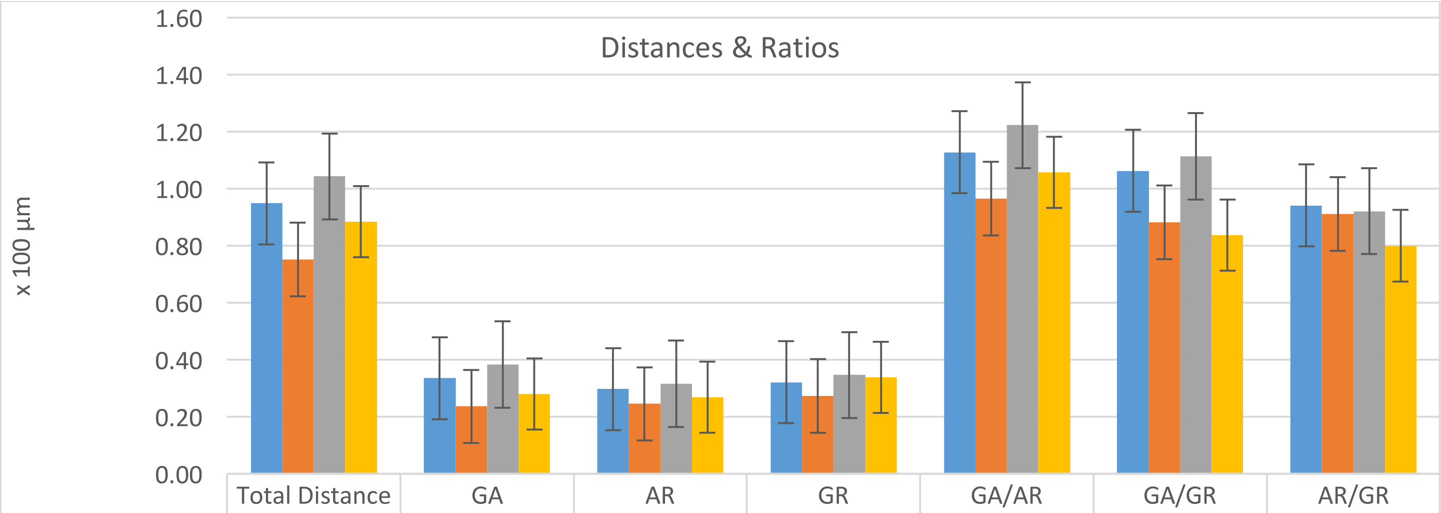

	Total Distance	GA	AR	GR	GA/AR	GA/GR	AR/GR
TT untreated	0.95	0.34	0.30	0.32	1.13	1.06	0.94
TT treated	0.75	0.24	0.25	0.27	0.96	0.88	0.91
GT untreated	1.04	0.38	0.32	0.35	1.22	1.11	0.92
GT treated	0.88	0.28	0.27	0.34	1.06	0.84	0.80

(ii) Total distances (GARG) for each genotype in response to treatment
Distances are displayed in units (1.0 unit = 100 pixels). Bars are mean ± SE.

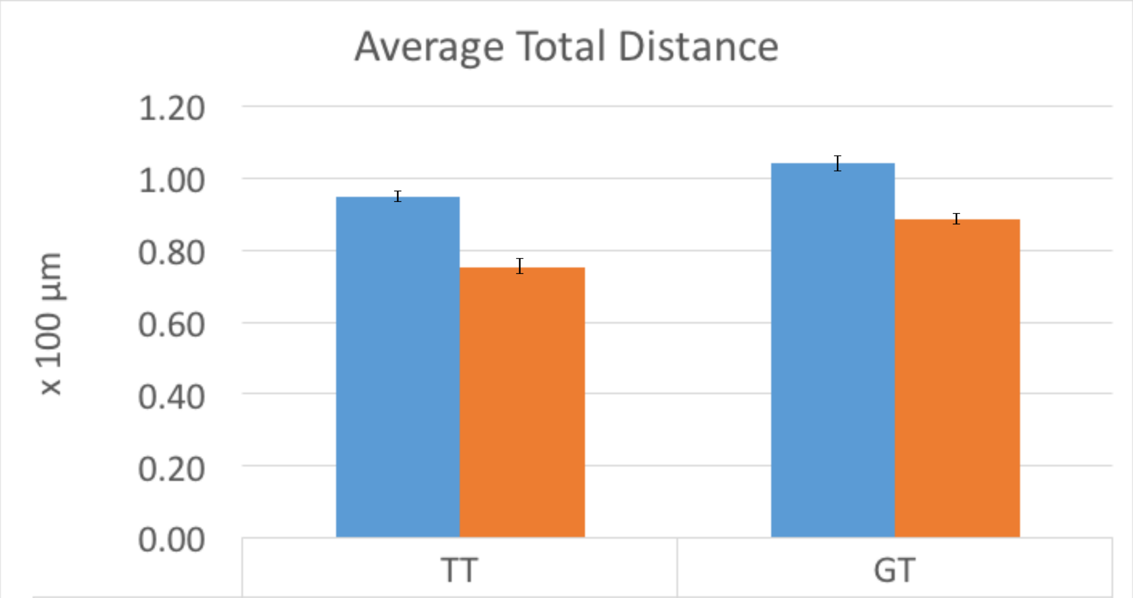

	TT	GT
Untreated	0.95	1.04
Treated	0.75	0.88

(iii) Angles between genes (mean ± SE of replicates are shown for 10 cell lines with the *FOXO3 TT* genotype and 10 cell lines with the *GT* genotype).

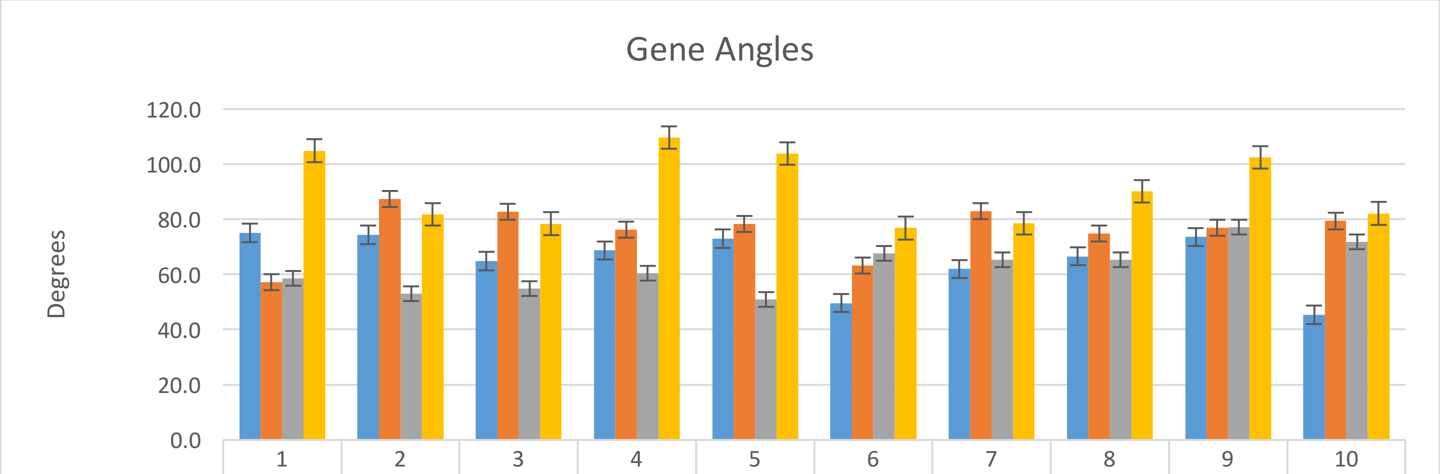

	1	2	3	4	5	6	7	8	9	10
TT untreated	75.1	74.4	64.8	68.7	73.0	49.6	62.0	66.5	73.5	45.3
TT treated	57.2	87.3	82.8	76.1	78.3	63.2	82.9	74.9	76.9	79.3
GT untreated	58.6	53.1	54.8	60.5	50.9	67.7	65.2	65.2	77.1	71.8
GT treated	104.8	81.8	78.4	109.7	103.9	76.8	78.5	90.2	102.4	82.1

(iv) Average angles between genes (mean ± SE)

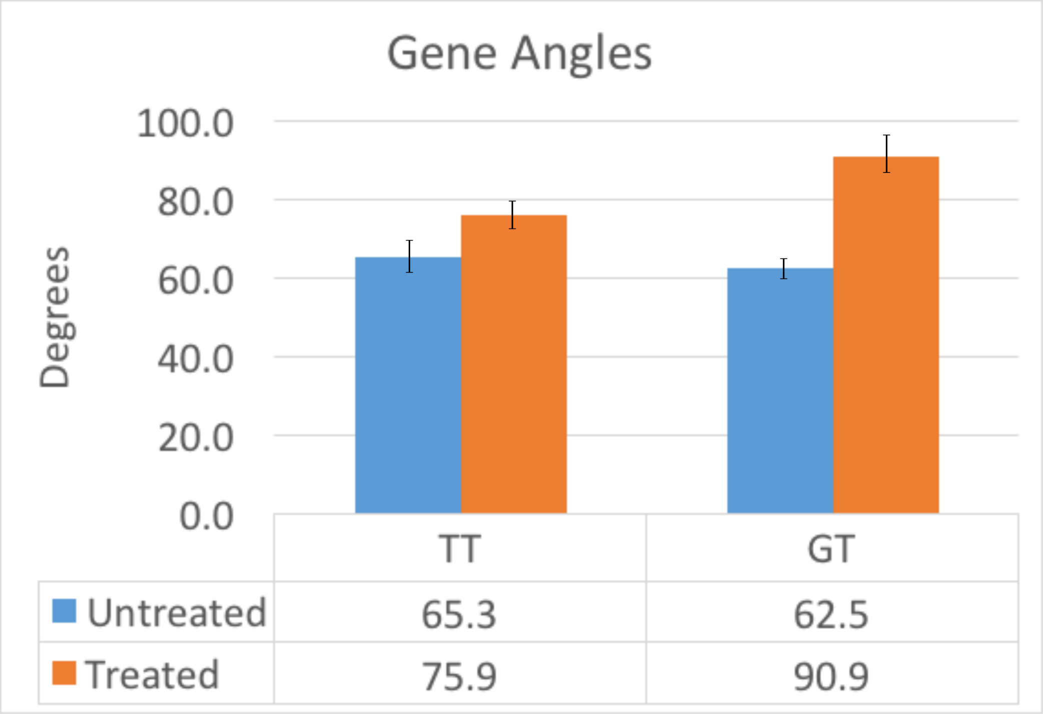

(v) Overall changes in gene relationships following treatment

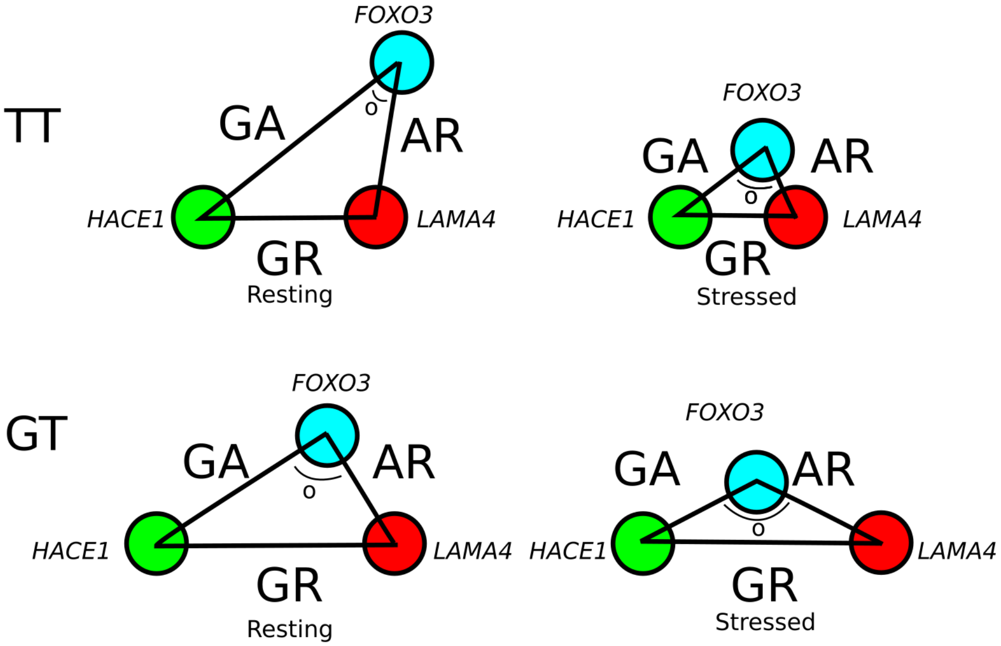

(c) *FOXO3* expression by genotype

(i) Average *FOXO3* mRNA level for resting vs. treated cells (mean ± SE) and comparing cells with *FOXO3 TT* vs. *GT* genotype.

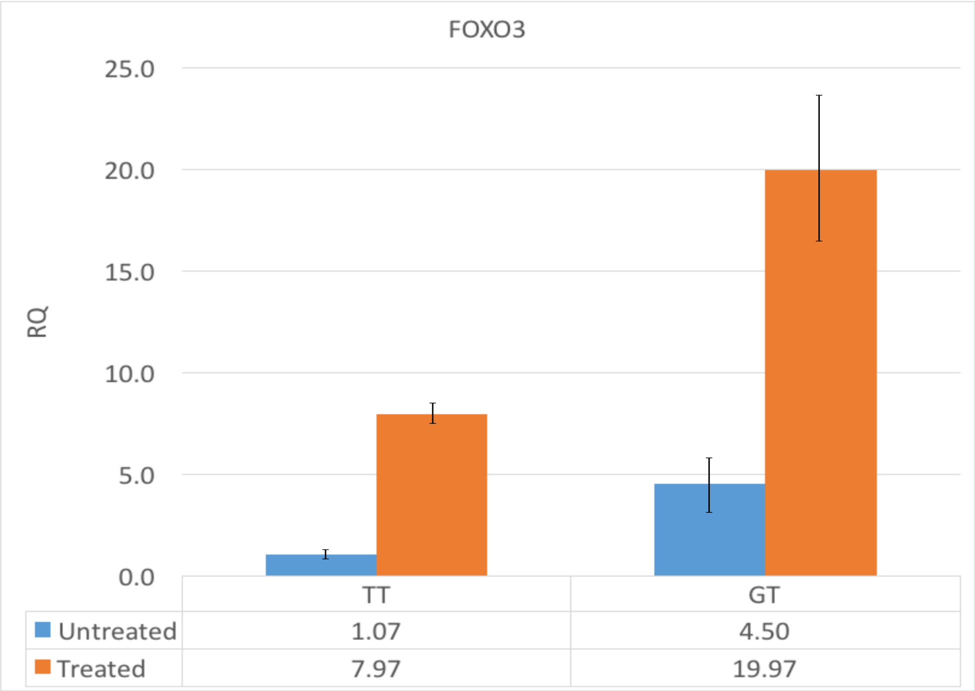

(ii) Average *HACE1* mRNA for resting vs. treated cells and comparing cells with *FOXO3 TT* vs. *GT* genotype (mean ± SD)

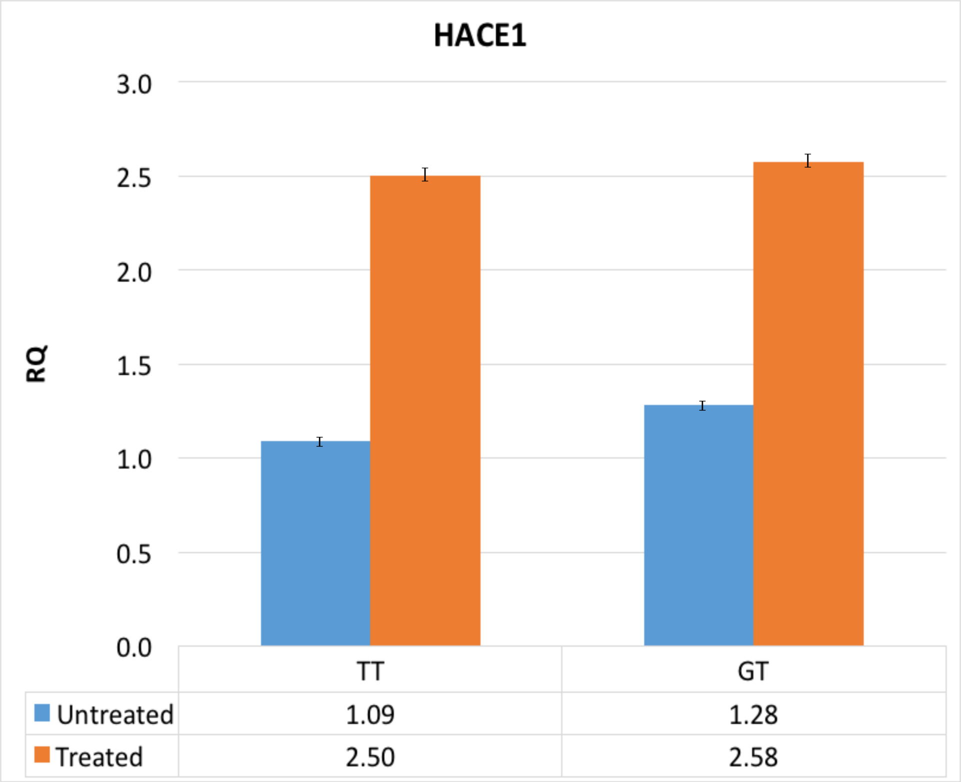

Fig. S5 shows results of FISH experiments using lymphoblast cell lines derived from 20 subjects who were the offspring of the long-lived Japanese American subjects used for the genetic association studies of *FOXO3* SNPs. We measured the distances between fluorescent signals for *HACE1* and *FOXO3* (GA), between *FOXO3* and *LAMA4* (AR), and between *HACE1* and *LAMA4* (GR). Early evaluation and quality control of the FISH probes suggested that relative gene position might affect expression. We therefore chose to also measure the angles between the three probes using *FOXO3* as the vertex (GAR).
(a) shows what measurements were made.
(b) (i) shows the mean distances, comparing paired untreated vs. treated cell lines from 10 subjects homozygous for the common allele of *FOXO3* (*TT*), and paired untreated vs. treated cell lines from 10 subjects heterozygous for the longevity-associated *G* allele (*GT* genotype). Ratios of distances were also compared in an effort to reduce cell-to-cell variability in nuclei diameter.
(b) (ii) shows total distance (i.e. circumference; GARG, mean±SE). Data were analyzed by *t*-test. While the differences between *TT* vs. *GT* genotypes were not significant (P=0.053), differences between treated and untreated were highly significant (P<0.0001).
(b) (iii) shows confirmation of this relationship by measuring the angles of the three genes to each other for 20 cell lines from different subjects with *TT* genotype (10 untreated and 10 treated) and 20 with *GT* genotype (10 untreated and 10 treated).
(b) (iv) shows mean±SE of data from (iii). Angles increased following treatment (P<0.0001). The results indicated that in the treated cells the three genes were more co-linear (in line with each other).
(b) (v) is a diagrammatic summary of the changes in gene positions that were seen for each genotype for untreated (resting) and treated (stressed) cells. It appeared that, upon activation, *FOXO3* became interposed between the two outlying genes, *HACE1* and *LAMA4*, and that the longevity genotype was already closer to that final position than the common genotype.
To summarize part (b):
• The total distance “GARG” was significantly shortened following treatment.
• The G-A-R angle was significantly increased following treatment.
• Less change was noted in cells carrying the *G* allele since *FOXO3* was more likely to already be in the “induced” orientation.
(c) shows the increase in *FOXO3* and *HACE1* expression in response to treatment of lymphoblast cell lines with H_2O_2 (part (i), P<0.0001 comparing expression and genotypes by *t*-test). There was no significant difference in expression of *HACE1* with respect to *FOXO3* genotype (part ii).

References

Hedrick PW (1987) Gametic disequilibrium measures: Proceed with caution. *Genetics* **117**, 331-342.
Zhou X, Maricque B, Xie M, Li D, Sundaram V, Martin EA, Koebbe BC, Nielsen C, Hirst M, Farnham P, Kuhn RM, Zhu J, Smirnov I, Kent WJ, Haussler D, Madden PA, Costello JF, Wang T (2011) The Human Epigenome Browser at Washington University. *Nat Methods* **8**, 989-990.
