# Supplementary material for: FOXO3 longevity interactome on chromosome 6
Source: Aging Cell. 2017 Jul 19;16(5):1016–25. doi: 10.1111/acel.12625 (PMC5595686; doi:10.1111/acel.12625)
Supplement: Supplementary file 2 — Table S1. FOXO3 SNPs evaluated in this study. Table S2. Transcription factors modified by variants. Table S3. Expression patterns of longevity‐associated transcription factors. Table S4. Genes on chromosome 6q21. Table S5. Expression patterns of genes on chromosome 6q21. Table S6. Published GWAS results for chromosome 6q21. Table S7. Amplification and sequencing primers. [file ACEL-16-1016-s002.docx]

**Supporting Information: Supplemental Tables**

**Suppl. Table S1. *FOXO3* SNPs evaluated in this study**

| **SNP ID** | **Position GRCh37.p13** | **MAF** | **Alleles** | **ORs** | **Significance** | **-LOG10(P)** | **TFBS** |
| --- | --- | --- | --- | --- | --- | --- | --- |
| rs12198276 | 108871964 | 0.133 | C/A | 0.754 | 0.104586 | 0.98 |  |
| rs768023 | 108876002 | 0.320 | A/G | 1.621 | 0.000707 | 3.15 | FOXOA/HDAC2 |
| rs58775446 | 108883685 | 0.058 | C/A | 1.340 | 0.185615 | 0.73 |  |
| rs9372188 | 108884734 | 0.050 | C/T | 1.340 | 0.273201 | 0.56 |  |
| rs9372189 | 108884891 | 0.068 | C/T | 1.340 | 0.273201 | 0.56 |  |
| rs1536057 | 108885622 | 0.163 | C/T | 1.704 | 0.000318 | 3.50 | NRF1/E2F/POU5F1 |
| rs72940674 | 108887557 | 0.067 | C/T | 1.340 | 0.273201 | 0.56 |  |
| rs2253310 | 108888592 | 0.233 | G/C | 1.632 | 0.000099 | 4.00 | TFCP2L1 |
| rs113412119 | 108889019 | 0.149 | C/T | 1.340 | 0.185615 | 0.73 |  |
| rs35214426 | 108889630 | 0.058 | T/C | 1.340 | 0.185615 | 0.73 |  |
| rs9372190 | 108889821 | 0.078 | T/G | 1.340 | 0.185615 | 0.73 |  |
| rs12055603 | 108892234 | 0.085 | A/G | 1.340 | 0.273201 | 0.56 |  |
| rs72940678 | 108893712 | 0.085 | C/G | 1.340 | 0.273201 | 0.56 |  |
| rs72940679 | 108895372 | 0.067 | A/G | 1.340 | 0.273201 | 0.56 |  |
| rs2490272 | 108895385 | 0.244 | T/C | 1.666 | 0.000990 | 3.00 |  |
| rs57099886 | 108895791 | 0.067 | G/A | 1.340 | 0.185615 | 0.73 |  |
| rs2802288 | 108896214 | 0.238 | G/A | 1.632 | 0.000100 | 4.00 | MYF |
| rs2883881 | 108897385 | 0.067 | A/G | 1.340 | 0.185615 | 0.73 |  |
| rs12200646 | 108897661 | 0.091 | G/A | 1.571 | 0.009700 | 2.01 |  |
| rs9384683 | 108899834 | 0.083 | G/T | 1.390 | 0.081481 | 1.09 |  |
| rs10499051 | 108902179 | 0.070 | A/G | 1.340 | 0.273201 | 0.56 |  |
| rs2802292 | 108908518 | 0.239 | G/T | 1.666 | 0.000058 | 4.24 |  |
| rs13220810 | 108913201 | 0.151 | T/C | 0.785 | 0.163250 | 0.79 |  |
| rs17069647 | 108917687 | 0.067 | T/A | 1.394 | 0.171612 | 0.77 |  |
| rs55758249 | 108919645 | 0.067 | A/G | 1.394 | 0.171612 | 0.77 |  |
| rs9386741 | 108919969 | 0.057 | A/G | 1.394 | 0.214888 | 0.67 |  |
| rs9372191 | 108923524 | 0.057 | C/T | 1.340 | 0.185615 | 0.73 |  |
| rs7775621 | 108924789 | 0.05 | A/G | 1.394 | 0.214888 | 0.67 |  |
| rs2802295 | 108926495 | 0.350 | A/G | 1.632 | 0.000099 | 4.00 |  |
| rs2764261 | 108927841 | 0.148 | A/G | 1.406 | 0.005678 | 2.25 |  |
| rs12192569 | 108933656 | 0.099 | C/A | 1.551 | 0.003953 | 2.40 |  |
| rs12192758 | 108933807 | 0.099 | G/A | 1.551 | 0.003953 | 2.40 |  |
| rs2764264 | 108934460 | 0.211 | T/C | 1.543 | 0.000471 | 3.33 | NKX3 |
| rs12202234 | 108939082 | 0.092 | C/G | 1.551 | 0.003953 | 2.40 | HNF3 |
| rs7341233 | 108940279 | 0.094 | T/C | 1.504 | 0.003953 | 2.40 |  |
| rs17598747 | 108941392 | 0.092 | A/G | 1.551 | 0.003953 | 2.40 |  |
| rs17069665 | 108941467 | 0.078 | A/G | 1.551 | 0.003201 | 2.49 | TFE |
| rs2022464 | 108945369 | 0.218 | C/A | 1.505 | 0.001384 | 2.86 |  |
| rs9285397 | 108951654 | 0.064 | C/T | 1.447 | 0.097174 | 1.01 |  |
| rs72942591 | 108951875 | 0.042 | C/G | 1.087 | 0.748362 | 0.13 |  |
| rs12213895 | 108951967 | 0.092 | T/A | 1.598 | 0.002802 | 2.55 | MEF2 |
| rs73763154 | 108955860 | 0.092 | T/C | 1.551 | 0.003953 | 2.40 |  |
| rs72942595 | 108957432 | 0.100 | G/A | 1.551 | 0.003953 | 2.40 |  |
| rs17310529 | 108957530 | 0.093 | T/C | 1.551 | 0.003953 | 2.40 |  |
| rs9486913 | 108960839 | 0.08 | C/G | 1.447 | 0.090804 | 1.04 |  |
| rs12202049 | 108968677 | 0.080 | G/A | 1.551 | 0.003953 | 2.40 |  |
| rs12209092 | 108970679 | 0.078 | A/G | 1.551 | 0.003953 | 2.40 |  |
| rs72944313 | 108973902 | 0.092 | C/T | 1.087 | 0.748362 | 0.13 |  |
| rs13217795 | 108974097 | 0.211 | C/T | 1.525 | 0.001174 | 2.93 |  |
| rs11153120 | 108974452 | 0.093 | C/A | 1.551 | 0.003953 | 2.40 |  |
| rs4946932 | 108974746 | 0.221 | C/A | 1.525 | 0.003953 | 2.40 |  |
| rs7772662 | 108977106 | 0.081 | G/A | 1.551 | 0.001174 | 2.93 |  |
| rs77603779 | 108977146 | 0.025 | C/T | 1.551 | 0.003953 | 2.40 |  |
| rs9400239 | 108977662 | 0.205 | C/T | 1.532 | 0.000837 | 3.08 |  |
| rs12212067 | 108981196 | 0.099 | T/G | 1.562 | 0.003953 | 2.40 | MZF1 |
| rs11153121 | 108982281 | 0.075 | C/T | 1.516 | 0.004861 | 2.31 |  |
| rs9398171 | 108983526 | 0.205 | T/C | 1.512 | 0.003988 | 2.40 | NR2F1/HNF4/HNF6 |
| rs12196996 | 108984066 | 0.093 | A/G | 1.516 | 0.004861 | 2.31 |  |
| rs2153960 | 108988183 | 0.215 | G/A | 1.485 | 0.002033 | 2.69 |  |
| rs3800226 | 108988594 | 0.078 | T/C | 1.551 | 0.003953 | 2.40 |  |
| rs73763159 | 108991685 | 0.092 | G/T | 1.516 | 0.004861 | 2.31 | BLIMP1/PRDM1 |
| rs3800229 | 108996963 | 0.174 | G/T | 1.486 | 0.002245 | 2.65 |  |
| rs3800230 | 108998127 | 0.141 | T/G | 1.422 | 0.028629 | 1.54 | FOXP1 |
| rs1935952 | 108998904 | 0.189 | G/C | 1.439 | 0.003311 | 2.48 | MZF1 |
| rs4946938 | 109020634 | 0.122 | T/C | 1.301 | 0.129058 | 0.89 |  |

A total of 65 single nucleotide polymorphisms (SNPs) were evaluated in 528 participants (187 cases + 341 controls) for longevity (living to ≥95 yrs.). Those SNPs having an odds ratio of ≥1.4 and a *P*-value of <0.05 are highlighted in green. Those SNPs that overlap with a functional feature (TFBS) are denoted. SNP ID is from the database “dbSNP”. Position is based on GRCh37.p13. MAF = minor allele frequency, Alleles are Major/Minor. OR = odds ratio; –LOG10(P) = negative log of the *P*-value; TFBS = transcription factor binding site.

**Suppl. Table S2. Transcription factors modified by variants.**

| **SNP ID #** | **Create Abolish** | **Factor** | **Name** | **Function** | **Expanded Function** | **GO** |
| --- | --- | --- | --- | --- | --- | --- |
| *rs768023* | abolish | FOXA | Forkhead box A | Glucose/fatty acid metabolism | FOXA (HNF3) is involved in embryonic development, establishment of tissue-specific gene expression and regulation of gene expression in differentiated tissues. Pioneer factor opening the compacted chromatin for other proteins through interactions with nucleosomal core histones and thereby replacing linker histones at target enhancer and/or promoter sites. | Transcription factor activity, sequence-specific DNA binding and transcription factor binding. Paralog of FOXA2 |
| *rs768023* | abolish | HDAC | Histone deacetylase | Histone modification | Control of cell proliferation and differentiation. Deacetylates p53 and modulates its effect on cell growth and apoptosis. | Transcription factor activity, sequence-specific DNA binding and transcription factor binding. |
| *rs1536057* | abolish | NRF1 | Nuclear respiratory factor 1 | Growth/respiration | Regulator of key metabolic genes regulating cellular growth and nuclear genes required for respiration, heme biosynthesis, and mitochondrial DNA transcription and replication. | RNA polymerase II core promoter proximal region sequence-specific DNA binding and transcriptional activator activity, |
| *rs1536057* | abolish | E2F | E2F transcription factor 1 | Proliferation vs. apoptosis | Cell cycle check point in response to DNA damage; regulated by RB | A multiprotein complex containing a retinoblastoma (Rb) family member. This complex is capable of repressing transcription of E2F-regulated genes in order to regulate cell cycle progression. |
| *rs1536057* | create | POU5F1 (OCT3/4) | POU domain, class 5, transcription factor 1 | Growth/differentiation; stem cell induction | Regulators of tissue-specific gene expression in lymphoid and pituitary differentiation and in early mammalian development and the expression of genes in insulin-secreting cells of the pancreas | Transcription factor |
| *rs2253310* | create | TFCP2L1 | Transcription factor CP2-like 1 | Growth/differentiation | Involved in regulation of the alpha-globin gene in erythroid cells. | Transcription factor |
| *rs2802288* | create | MYF | Myogenic factor | Muscle homeostasis | Plays a role in muscle stem cell maintenance and differentiation | Transcription factor |
| *rs2764264* | abolish | NKX3 | NK3 homeobox 2 | Growth/differentiation | Plays an essential role in craniofacial development and in ossification of the vertebral column | Transcription factor |
| *rs12202234* | abolish | HNF3/FOXOA1 | Forkhead box A | Glucose/fatty acid metabolism | Hepatocyte nuclear factors are transcriptional activators for liver-specific transcripts. Involved in the development of multiple endoderm-derived organ systems such as liver, pancreas, lung and prostate. | Transcription factor; steroid hormone receptor |
| *rs17069665* | abolish | TFE (MITF) | Microphthalmia-associated transcription factor | Growth/differentiation | Regulates the expression of genes with essential roles in cell differentiation, proliferation and survival. | Transcription factor |
| *rs12213895* | create | MEF2 | myocyte enhancer factor 2 | Muscle homeostasis | Activates many muscle-specific, growth factor-induced, and stress-induced genes. Involved in cellular processes, including muscle development, neuronal differentiation, cell growth control, and apoptosis. | Transcription factor |
| *rs12212067* | create | MZF1 | Myeloid zinc finger 1 | Hematopoietic maintenance | May be one regulator of transcriptional events during hemopoietic development. | Transcription factor |
| *rs9398171* | create | NR2F1 | Nuclear receptor subfamily 2, group F, member 1 | Glucose/fatty acid metabolism | Involved in developmental biology and Oct4 in mammalian ESC pluripotency. | Transcription factor; steroid hormone receptor |
| *rs9398171* | create | HNF4 | Hepatocyte nuclear factor 4 | Glucose/fatty acid metabolism | Regulates the expression of several hepatic genes, liver, kidney, and intestine. Involved in monogenic autosomal dominant non-insulin-dependent diabetes mellitus type I. | Transcription factor; steroid hormone receptor |
| *rs9398171* | abolish | HNF6 (ONECUT1) | Hepatocyte nuclear factor 6 | Glucose/fatty acid metabolism | Stimulates transcription of liver-expressed genes, and antagonizes glucocorticoid-stimulated gene transcription; MODY | Transcription factor; steroid hormone receptor |
| *rs73763159* | abolish | PRDM1 (BLIMP1) | PR domain containing 1, with ZNF domain | Hematopoietic maintenance | Repressor of beta-interferon gene expression | Transcription factor |
| *rs3800230* | abolish | FOXP1 | Forkhead box P1 | Growth/differentiation | Regulation of tissue- and cell type-specific gene transcription during both development and adulthood; tumor suppressor; neuronal stem cell development. Stimulates expression of transcription factors that are required for pluripotency and decreases expression of differentiation-associated genes. | Transcription factor |
| rs1935952 | abolish | MZF1 | Myeloid zinc finger 1 | Hematopoietic maintenance | May be one regulator of transcriptional events during hemopoietic development. | Transcription factor |

**Suppl. Table S2. Transcription factors modified by variants.**

The 13 SNPs modify 18 transcription factor binding sites shown here, along with their functions. SNP ID# = variant name from the database dbSNP, Create/Abolish refers to whether the minor allele creates or abolishes a binding site, Factor = transcription factor, Name = name of transcription factor, Function = short description of function, Expanded Function = additional functional information, GO = Gene Ontology summary.

**Suppl. Table S3. Expression patterns of longevity-associated transcription factors.**

**
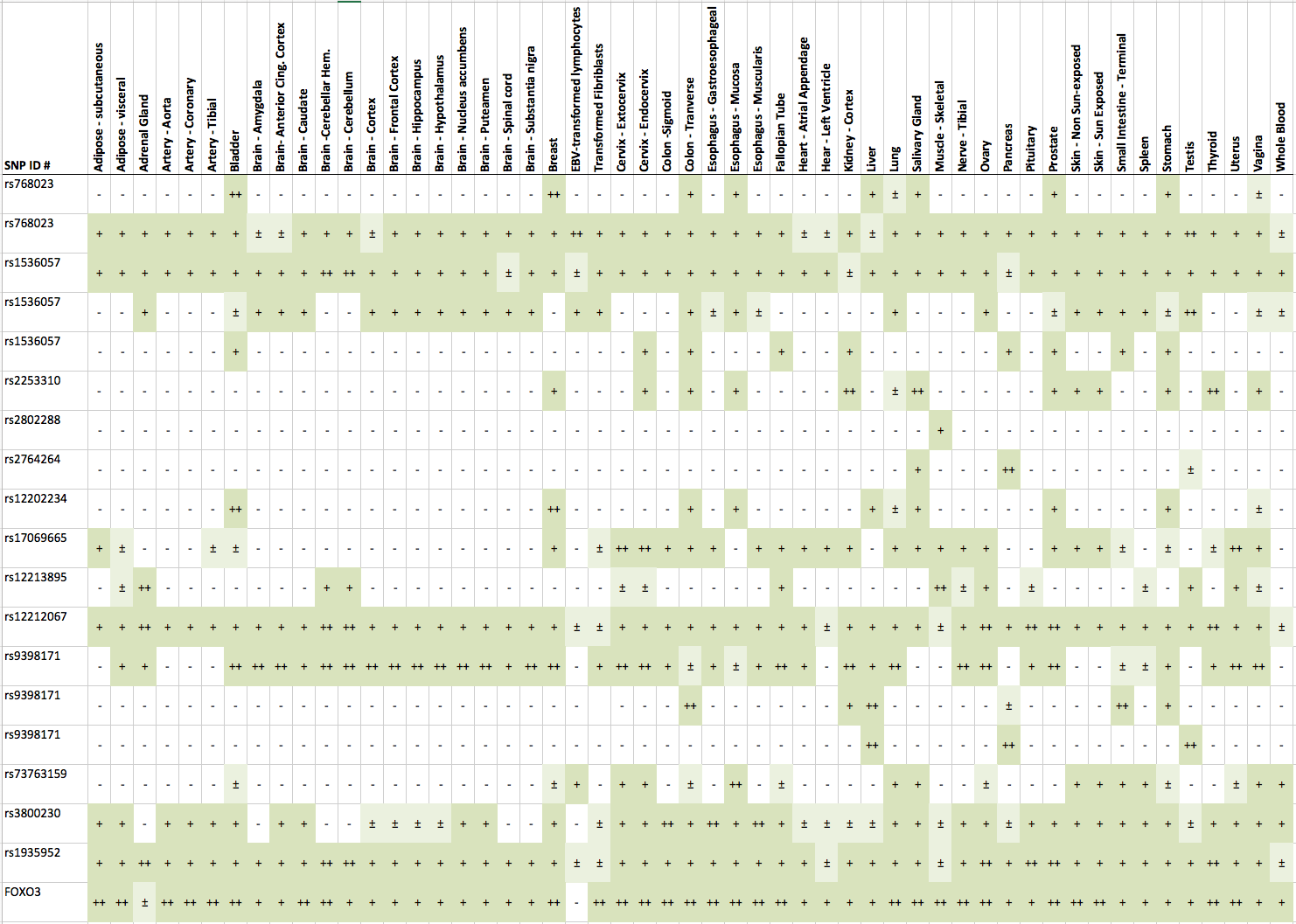
**

Table S3 shows are the expression pattern distributions for the18 transcription factors from Table S2 across 53 tissues. *FOXO3* is shown for comparison and is expressed in the majority of tissues.

– less than 2 RPKM

± less than 5 RPKM

+ 5–10 RPKM

++ > 10 RPKM

RPKM = reads per kb per million reads, and is a method for defining gene expression levels from the GTEx database. From GTES, gene expression values for all samples from a given tissue were normalized using the following procedure:

- Genes were selected based on expression thresholds of >0.1 RPKM in at least 10 individuals and ≥ 6 reads in at least 10 individuals.
- Expression values were quantile normalized to the average empirical distribution observed across samples.
- For each gene, expression values were inverse quantile normalized to a standard normal distribution across samples.

**Suppl. Table S4. Genes on chromosome 6q21**

| **Gene** | **Position** | **Distance (bp)** | **Name** | **GO** | **Function** |
| --- | --- | --- | --- | --- | --- |
| *HACE1* | 105307794 | –3573231 | HECT domain and ankyrin repeat containing E3 ubiquitin protein ligase 1 | The protein contains a HECT domain and ankyrin repeat-containing ubiquitin ligase. Ligase activity and Rab GTPase binding. | Among its related pathways are immune system and SMAD signaling network. An important paralog of this gene is AREL1. Diseases associated with HACE1 include neuroblastoma. |
| *LIN28B* |  |  | Lin-28 homolog B | Nucleic acid binding and RNA binding. | Diseases associated with LIN28B include oral cavity cancer and neuroblastoma. Among its related pathways are Validated targets of C-MYC transcriptional activation and cardiac progenitor differentiation. |
| *BVES* | 105544698 | –3,336,327 | Blood vessel epicardial substance | Structural molecule activity and cAMP binding. | A member of the POP family of proteins containing three putative transmembrane domains. This gene is expressed in cardiac and skeletal muscle and may play an important role in development of these tissues. Involved in skeletal muscle and heart development. Diseases associated with BVES include tetralogy of fallot. The mouse ortholog may be involved in the regeneration of adult skeletal muscle and may act as a cell adhesion molecule in coronary vasculogenesis. Also involved in striated muscle regeneration and repair and in the regulation of cell spreading. |
| *POPDC3* | 105606155 | –3,274,870 | Popeye domain containing 3 | Integral component of membrane, cAMP binding |  |
| *PREP* | 105,725,442 | –3,155,583 | Prolyl endopeptidase | Serine-type endopeptidase activity and serine-type exopeptidase activity. | Cytosolic prolyl endopeptidase that cleaves peptide bonds on the C-terminal side of prolyl residues within peptides that are up to approximately 30 amino acids long. Prolyl endopeptidases have been reported to be involved in the maturation and degradation of peptide hormones and neuropeptides. |
| *PRDM1* | 106546736 | –2,334,289 | PR domain zinc finger protein 1 | Positive regulation of B cell differentiation, negative regulation of transcription from RNA polymerase II promoter, intestinal epithelial cell development. | Repressor of beta-interferon; NF-kappaB signaling and direct p53 effectors, drives the maturation of B-lymphocytes into Ig secreting cell. |
| *ATG5* | 106764141 | –2,116,884 | Autophagy protein 5 | Membrane trafficking regulatory protein, autophagosome assembly, mitophagy. | Autophagy. |
| *AIM1* | 106959729 | –1,921,296 | Absent In melanoma 1 | Carbohydrate binding. | May function as suppressor of malignant melanoma. It may exert its effects through interactions with the cytoskeleton. Diseases associated with AIM1 include albinism, oculocutaneous, type iv and melanoma. |
| *RTN4IP1* | 107077373 | –1,803,652 | Reticulon-4-interacting protein 1, mitochondrial | Oxidoreductase activity. | This gene encodes a novel mitochondrial protein that interacts with reticulon 4, which is a potent inhibitor of regeneration following spinal cord injury. The interaction of reticulon 4 with mitochondrial proteins may provide insight into the mechanisms for reticulon-induced inhibition of neurite growth. |
| *QRSL1* | 107077440 | –1,803,585 | Glutamyl-tRNA(Gln) amidotransferase subunit A, mitochondrial | Regulation of protein stability, mitochondrial translation. | Allows the formation of correctly charged Gln-tRNA(Gln) through the transamidation of misacylated Glu-tRNA(Gln) in the mitochondria. The reaction takes place in the presence of glutamine and ATP through an activated gamma-phospho-Glu-tRNA(Gln). |
| *C6orf203* | 107349417 | –1,531,608 | Chromosome 6 open reading fame 203 | Mitochondrion. | Contains a CTCF binding site within gene. |
| *BEND3* | 107435636 | –1,445,389 | BEN domain-containing protein 3 | Chromatin silencing at rDNA, nuclear heterochromatin, histone modifications. | Transcriptional repressor which associates with the NoRC (nucleolar remodeling complex) complex and plays a key role in repressing rDNA transcription. The sumoylated form modulates the stability of the NoRC complex component BAZ2A/TIP5 by controlling its USP21-mediated deubiquitination (PubMed:21914818, PubMed:26100909). Binds to unmethylated major satellite DNA and is involved in the recruitment of the polycomb repressive complex 2 (PRC2) to major satellites. |
| *PDSS2* | 107780779 | –1,100,246 | Decaprenyl-diphosphate synthase subunit 2 | Ubiquinone biosynthetic process, mitochondrial matrix | The protein encoded by this gene is an enzyme that synthesizes the prenyl side-chain of coenzyme Q, or ubiquinone, one of the key elements in the respiratory chain. The gene product catalyzes the formation of all trans-polyprenyl pyrophosphates from isopentyl diphosphate in the assembly of polyisoprenoid side chains, the first step in coenzyme Q biosynthesis. Defects in this gene are a cause of coenzyme Q10 deficiency. |
| *SOBP* | 107811316 | –1,069,709 | Sine oculis-binding protein homolog | SUMO polymer binding, inner ear morphogenesis. | Nuclear zinc finger protein that is involved in development of the cochlea. Defects in this gene have also been linked to intellectual disability. |
| *SCML4* | 108145521 | –735,504 | Sex comb on midleg-like protein 4 | Regulation of transcription, DNA-templated. | Putative polycomb group (PcG) protein. PcG proteins act by forming multiprotein complexes, which are required to maintain the transcriptionally repressive state of homeotic genes throughout development (By similarity). |
| *SEC63* | 108188959 | –692,066 | Translocation protein SEC63 homolog | Chaperone, endoplasmic reticulum membrane, IRE1-mediated unfolded protein response, multicellular organism aging. | Protein degradation. |
| *OSTM1* | 108395941 | –485,084 | Osteopetrosis-associated transmembrane protein 1 | Transport of glucose and other sugars, bile salts and organic acids, metal ions and amine compounds and Ion channel transport. | This gene encodes a protein that may be involved in the degradation of G proteins via the ubiquitin-dependent proteasome pathway. Defects in this gene may cause the autosomal recessive, infantile malignant form of osteopetrosis. Diseases associated with OSTM1. include osteopetrosis, autosomal recessive 5 and infantile osteopetrosis with neuroaxonal dysplasia. |
| *NR2E1* | 108489226 | –391,799 | Nuclear receptor subfamily 2 group E member 1 | Nuclear hormone receptor; receptor; nucleic acid binding. cerebral cortex neuron differentiation, somatic stem cell population maintenance, histone deacetylase binding | Orphan nuclear receptor. |
| *SNX3* | 108582464 | –298,561 | Sorting nexin-3 | membrane trafficking regulatory protein, Wnt signaling pathway. | Protein trafficking. |
| *LACE1* | 108616097 | –264,928 | Lactation elevated protein 1 | hydrolase, ATP binding, mitochondrion. | Mitochondrial protein homeostasis; cell growth. |
| *FOXO3* | 108881025 | 0 | Forkhead box protein O3 | transcription factor; DNA binding protein. | Transcription factor; survival/senescence; positive regulation of apoptotic process response to oxidative stress, stem cell maintenance; positive regulation of erythrocyte differentiation; DNA damage response, signal transduction by p53 class mediator; regulation of neural precursor cell proliferation. |
| *LINC00222* | 109072856 | 191,831 | Long intergenic non-protein coding RNA 222 | - | Long non-coding RNA; chromatin organization/gene regulation. |
| *ARMC2* | 109169618 | 288,593 | Armadillo repeat-containing protein 2 | Protein binding. | Armadillo repeat containing protein promotes protein interaction. |
| *SESN1* | 109330758 | 449,733 | Sestrin-1 | Cellular response to amino acid stimulus; regulation of response to reactive oxygen species; cellular oxidant detoxification, negative regulation of TORC1 signaling. | Response to DNA damage/oxidative damage; cell growth. |
| *CEP57L1* | 109416355 | 535,330 | Centrosomal protein CEP57L1 | Mitotic sister chromatid segregation; microtubule binding, gamma-tubulin binding. | Centrosomal protein which may be required for microtubule attachment to centrosomes. Similar to translokin. |
| *CD164* | 109703762 | 822,737 | Sialomucin core protein 24 | Negative regulation of cell adhesion; negative regulation of cell proliferation; muscle organ development; hemopoiesis. | Sialomucin that may play a key role in hematopoiesis by facilitating the adhesion of CD34(+) cells to the stroma and by negatively regulating CD34(+)CD38(lo/–) cell proliferation. Modulates the migration of umbilical cord blood CD133+ cells and this is mediated through the CXCL12/CXCR4 axis. May play an important role in prostate cancer metastasis and the infiltration of bone marrow by cancer cells. Promotes myogenesis by enhancing CXCR4-dependent cell motility. Positively regulates myoblast migration and promotes myoblast fusion into myotubes (by similarity). |
| *SMPD2* | 109761930 | 880,905 | Sphingomyelin phosphodiesterase 2 | Positive regulation of ceramide biosynthetic process; sphingomyelin phosphodiesterase activity; response to mechanical stimulus. | This gene encodes a protein which was identified initially as a sphingomyelinase based on sequence similarity between bacterial sphingomyelinases and a yeast protein. Subsequent studies showed that its biological function is less likely to be as a sphingomyelinase and instead as a lysophospholipase. |
| *PPIL6* | 109762374 | 881,349 | Peptidyl-prolyl cis-trans isomerase-like 6 | Peptidyl-prolyl cis-trans isomerase activity; protein folding. | PPIases accelerate the folding of proteins. The encoded enzyme catalyzes the cis-trans isomerization of proline imidic peptide bonds in oligopeptides. |
| *MICAL1* | 109787171 | 906,146 | Protein-methionine sulfoxide oxidase MICAL1 | Actin filament depolymerization; negative regulation of cysteine-type endopeptidase activity involved in apoptotic process. | This gene encodes an enzyme that oxidizes methionine residues on actin, thereby promoting depolymerization of actin filaments. This protein interacts with and regulates signaling by NEDD9/CAS-L (neural precursor cell expressed, developmentally down-regulated 9). |
| *ZBTB24* | 109804440 | 923,415 | Zinc finger and BTB domain-containing protein 24 | Hematopoietic progenitor cell differentiation. | This gene encodes a protein similar to a protein in rodents which is induced by bone morphogenic protein 2 in vitro. Diseases associated with ZBTB24 include immunodeficiency-centromeric instability-facial anomalies syndrome-2 and immunodeficiency, centromere instability and facial anomalies syndrome. |
| *AK9* | 110012415 | 1,131,390 | Adenylate kinase 9 | Nucleobase-containing compound kinase activity; ATP-binding. | Involved in maintaining the homeostasis of cellular nucleotides by catalyzing the interconversion of nucleoside phosphates. Has both nucleoside monophosphate and diphosphate kinase activities. |
| *FIG4* | 110012423 | 1,131,398 | Polyphosphoinositide phosphatase | Endoplasmic reticulum; positive regulation of neuron projection development; myelin assembly; recycling endosome. | The yeast homolog, Sac1p, is involved in the regulation of various phosphoinositides, and affects diverse cellular functions such as actin cytoskeleton organization, golgi function, and maintenance of vacuole morphology. Membrane-bound phosphoinositides function as signaling molecules and play a key role in vesicle trafficking in eukaryotic cells. |
| *GPR6* | 110299458 | 1,418,433 | G protein-coupled receptor 6 | G-protein coupled receptor activity; signal transduction. | Orphan receptor with constitutive G(s) signaling activity that activate cyclic AMP. Promotes neurite outgrowth and blocks myelin inhibition in neurons. |
| *WASF1* | 110501207 | 1,620,182 | Wiskott-Aldrich syndrome protein family member 1 | Actin binding and Rac GTPase binding. | Downstream effector molecule involved in the transmission of signals from tyrosine kinase receptors and small GTPases to the actin cytoskeleton. Promotes formation of actin filaments. Part of the WAVE complex that regulates lamellipodia formation. The WAVE complex regulates actin filament reorganization via its interaction with the Arp2/3 complex. |
| *CDC40* | 110501623 | 1,620,598 | Pre-mRNA-processing factor 17 | mRNA splicing, via spliceosome; | The protein encoded by this gene is found to be essential for the catalytic step II in pre-mRNA splicing process. It is found in the spliceosome, and contains seven WD repeats, which function in protein-protein interactions. This protein has a sequence similarity to yeast Prp17 protein, which functions in two different cellular processes: pre-mRNA splicing and cell cycle progression. It suggests that this protein may play a role in cell cycle progression. |
| *METTL24* | 110567131 | 1,686,106 | Methyltransferase like 24 | methyltransferase activity | ? |
| *DDO* | 110736753 | 1,855,728 | D-aspartate oxidase | D-amino-acid oxidase activity; cofactor binding | The protein encoded by this gene is a peroxisomal flavoprotein that catalyzes the oxidative deamination of D-aspartate and N-methyl D-aspartate. |
| *SLC22A16* | 110797844 | 1,916,819 | Solute carrier family 22 member 16 | Amine transmembrane transporter activity; carnitine transmembrane transporter activity | This gene encodes a member of the organic zwitterion transporter protein family that transports carnitine. |
| *CDK19* | 111136412 | 2,255,387 | Cyclin-dependent kinase 19 | Cyclin-dependent protein serine/threonine kinase activity; positive regulation of apoptotic process; positive regulation of inflammatory response; cellular response to lipopolysaccharide | This gene encodes a protein that is one of the components of the mediator co-activator complex. The mediator complex is a multi-protein complex required for transcriptional activation by DNA binding transcription factors of genes transcribed by RNA polymerase II. |
| *AMD1* | 111195987 | 2,314,962 | S-adenosylmethionine decarboxylase proenzyme | Polyamine metabolic process; spermine biosynthetic process | Essential for biosynthesis of the polyamines spermidine and spermine. Promotes maintenance and self-renewal of embryonic stem cells by maintaining spermine levels (by similarity). |
| *GTF3C6* | 111279762 | 2,398,737 | General transcription factor 3C polypeptide 6 | Transcription from RNA polymerase III promoter | Involved in RNA polymerase III-mediated transcription. Integral, tightly associated component of the DNA-binding TFIIIC2 subcomplex that directly binds tRNA and virus-associated RNA promoters. |
| *RPF2* | 111303290 | 2,422,265 | Ribosome production factor 2 homolog | Maturation of LSU-rRNA from tricistronic rRNA transcript (SSU-rRNA, 5.8S rRNA, LSU-rRNA); poly(A) RNA binding and rRNA binding. | RPF2 (ribosome production factor 2 homolog) is a protein coding gene. |
| *SLC16A10* | 111408780 | 2,527,755 | Monocarboxylate transporter 10 | amino acid transmembrane transporter activity; thyroid hormone transport. | Transport of glucose and other sugars, bile salts and organic acids, metal ions and amine compounds and Thyroid hormone signaling pathway. |
| *MFSD4B* (*KIAA1919*) | 111580481 | 2,699,456 | Sodium-dependent glucose transporter 1 | carbohydrate transport; sodium ion transport. | Transport of glucose and other sugars, bile salts and organic acids, metal ions and amine compounds. |
| *TRAF3IP2* | 111804674 | 2,923,649 | Adapter protein CIKS | B cell apoptotic process; immunoglobulin secretion; positive regulation of I-kappaB kinase/NF-kappaB signaling. | Regulates response to cytokines. |
| *REV3L* | 111804918 | 2,923,893 | DNA polymerase zeta catalytic subunit | DNA-directed DNA polymerase activity . | Interacts with MAD2L2 to form the error prone DNA polymerase zeta involved in translesion DNA synthesis. |
| *FYN* | 112194655 | 3,313,630 | FYN protein | Non-receptor tyrosine protein kinase; non-receptor tyrosine protein kinas.e | Oncogene, cell growth. |
| *WISP3* | 112375370 | 3,494,345 | WNT1-inducible-signaling pathway protein 3 | Growth factor; signal transduction; regulation of cell growth; negative regulation of cell death; insulin-like growth factor binding. | Skeletal growth and cartilage homeostasis. |
| *TUBE1* | 112408751 | 3,527,726 | Tubulin Epsilon 1 | GTP binding and structural constituent of cytoskeleton. | Localizes to the centriolar sub-distal appendages that are associated with the older of the two centrioles after centrosome duplication. This protein plays a central role in organization of the microtubules during centriole duplication. |
| *LAMA4* | 112575917 | 3,694,892 | Laminin subunit alpha-4 | Receptor; extracellular matrix linker protein; extracellular matrix organization; regulation of cell adhesion/migration. | Cell adhesion, differentiation, migration, signaling. |

Table S4 lists the genes located on the 7,268,123 bp segment of chromosome 6q21 that are physically linked to each other by CTCF chromatin looping. Gene = gene symbol; Position = position on chromosome 6 based on GRCh37.p13; Distance = distance from the *FOXO3* promoter; Name = name of gene; GO = Gene Ontology summary; Function = accepted functions based on “GeneCards” and OMIM (Online Mendelian Inheritance in Man).

**
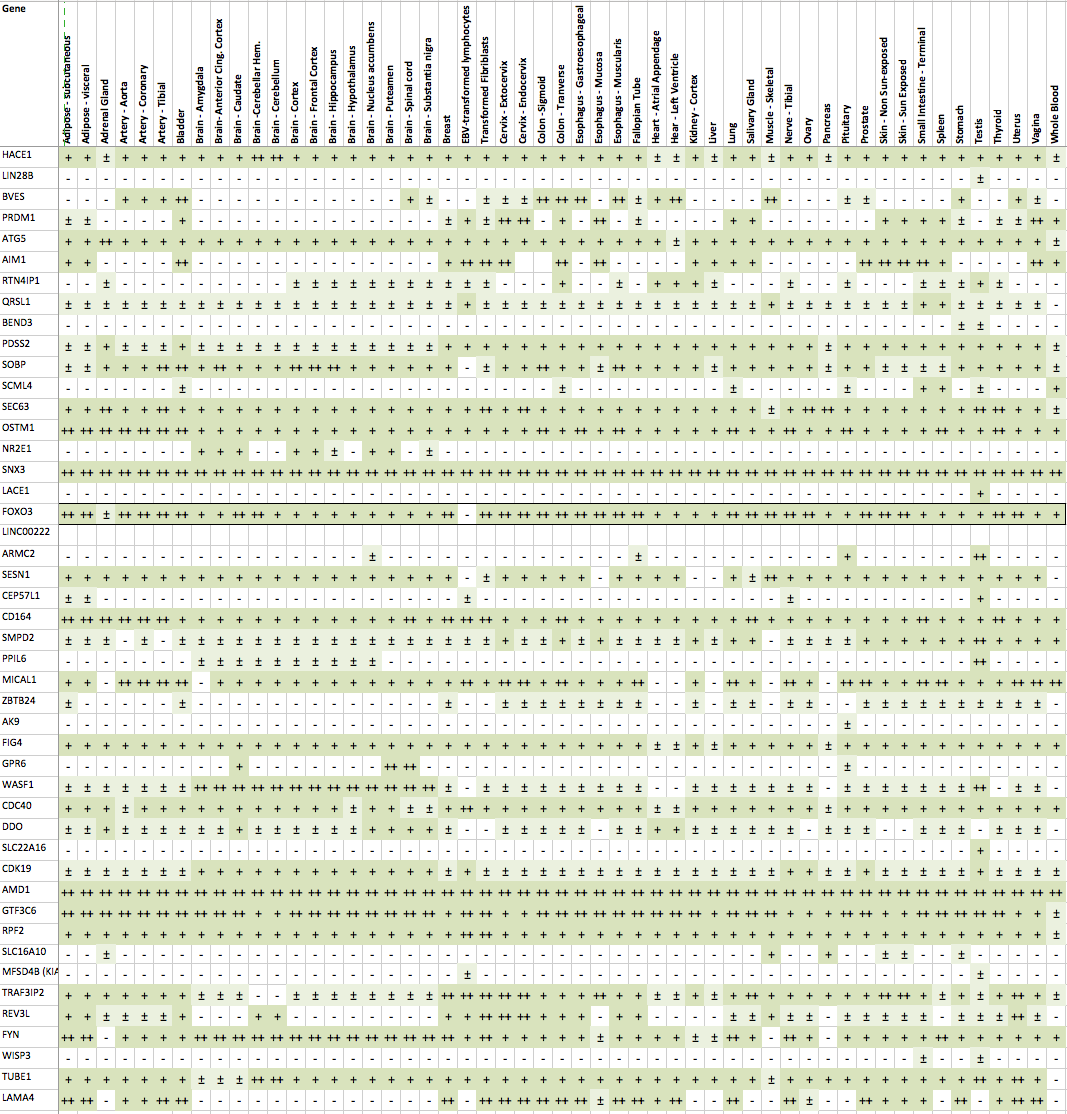
Suppl. Table S5. Expression patterns of genes on chromosome 6q21**

Table S5 shows the expression pattern distributions of the 46 genes from Table S4 in 53 tissues. *FOXO3* is shown (bracketed) for comparison and is expressed in the majority of tissues.

– less than 2 RPKM

± less than 5 RPKM

+ 5–10 RPKM

++ > 10 RPKM

RPKM = reads per kb per million reads, and is a method for defining gene expression levels from the GTEX database. From GTES, gene expression values for all samples from a given tissue were normalized using the following procedure:

- Genes were selected based on expression thresholds of >0.1 RPKM in at least 10 individuals and ≥6 reads in at least 10 individuals.
- Expression values were quantile normalized to the average empirical distribution observed across samples.
- For each gene, expression values were inverse quantile normalized to a standard normal distribution across samples.

**Suppl. Table S6. Published GWAS results for chromosome 6q21**

| **Gene** | **SNP ID** | **Location** | **Phenotype** | **Data source:** | ***P*-Value (–log10)** | **PubMed ID** | **Relevant Phenotype** |
| --- | --- | --- | --- | --- | --- | --- | --- |
| *HACE1* | *rs4336470* | 104732910 | Neuroblastoma | dbGaP | 4.1 | 23222812 | Cancer |
|  | *rs9322817* | 104784358 | Thyrotropin | dbGaP | 5.2 | 17903292 | Endocrine function |
|  | *rs9322817* | 104784358 | Thyrotropin | NHGRI GWAS catalog | 5.2 | 17903292 | Endocrine function |
| *LIN28B* | *rs17065417* | 104958399 | Neuroblastoma | dbGaP | 4.2 | 23222812 | Cancer |
|  | *rs314277* | 104959787 | Body height | NHGRI GWAS catalog | 8.0 | 18391950 | Growth |
|  | *rs314277* | 104959787 | Menarche | NHGRI GWAS catalog | 12.5 | 19448621 | Endocrine function |
|  | *rs314277* | 104959787 | Body height | NHGRI GWAS catalog | 5.7 | 20303062 | Growth |
|  | *rs314276* | 104960124 | Menarche | NHGRI GWAS catalog | 15.4 | 19448623 | Endocrine function |
|  | *rs314268* | 104970103 | Body height | dbGaP | 5.0 |  | Growth |
|  | *rs314268* | 104970103 | Body height | dbGaP | 5.1 |  | Growth |
|  | *rs314268* | 104970103 | Waist circumference | dbGaP | 7.6 |  | Growth |
|  | *rs314268* | 104970103 | Body height | NHGRI GWAS catalog | 6.1 | 18391951 | Growth |
|  | *rs369065* | 104996183 | Body height | dbGaP | 4.4 |  | Growth |
|  | *rs369065* | 104996183 | Waist circumference | dbGaP | 7.4 |  | Growth |
|  | *rs9377684* | 104997489 | Waist circumference | dbGaP | 5.2 |  | Growth |
|  | *rs9377684* | 104997489 | Waist circumference | dbGaP | 4.1 |  | Growth |
| *PRDM1* | *rs7746082* | 105987394 | Crohn disease | NHGRI GWAS catalog | 9.7 | 18587394 | Inflammation |
|  | *rs6911490* | 106074152 | Colitis, ulcerative | NHGRI GWAS catalog | 8.0 | 21297633 | Inflammation |
|  | *rs548234* | 106120159 | Lupus erythematosus, systemic | NHGRI GWAS catalog | 11.3 | 19838193 | Inflammation |
|  | *rs11152965* | 106123668 | Lipids | dbGaP | 4.4 |  | Endocrine function |
| *AIM1* | *rs783396* | 106539495 | Stroke | dbGaP | 5.0 | 17434096 | Ischemic stroke |
|  | *rs783396* | 106539495 | Stroke | NHGRI GWAS catalog | 5.0 | 17434096 | Ischemic stroke |
| *SOBP* | *rs699699* | 107621148 | Respiratory function tests | dbGaP | 5.0 | 17903307 | Growth |
|  | *rs1028498* | 107653874 | Body weights and measures | dbGaP | 5.5 | 17903300 | Growth |
|  | *rs1028498* | 107653874 | Body weights and measures | dbGaP | 5.5 | 17903300 | Growth |
|  | *rs7751374* | 107660652 | Stroke | dbGaP | 3.2 |  | Ischemic stroke |
| *FOXO3* | *rs9480865* | 108595370 | Multiple sclerosis | NHGRI GWAS catalog | 5.2 | 19010793 | Insulin response/growth |
|  | *rs7341233* | 108619077 | Creatinine | dbGaP | 4.2 |  | metabolites |
|  | *rs9285397* | 108630452 | Electrocardiography | dbGaP | 6.4 | 17903306 | Heart rate |
|  | *rs6911407* | 108545828 | Monocytes | dbGaP | 4.0 |  | Hematopoiesis |
|  | *rs2153960* | 108666981 | Insulin-like growth factor I | NHGRI GWAS catalog | 6.3 | 21216879 | Insulin response/growth |
|  | *rs13220810* | 108591998 | Blood pressure | dbGaP | 4.2 |  | Blood pressure |
|  | *rs2802292* | 108587315 | Blood pressure | KHHP |  | 26476085 | Blood pressure |
| *LINCOO2* | *rs568064* | 108788893 | Hypertrophy, left ventricular | dbGaP | 4.4 |  | Muscle growth |
|  | *rs1268128* | 108795288 | Hypertrophy, left ventricular | dbGaP | 4.5 |  | Muscle growth |
| *ARMC2* | *rs13219800* | 108919359 | Hypertrophy, left ventricular | dbGaP | 4.9 |  | Muscle growth |
|  | *rs13195372* | 108938172 | Diabetes mellitus | dbGaP | 4.2 |  | Diabetes |
|  | *rs2798641* | 108946847 | Respiratory function tests | NHGRI GWAS catalog | 8.1 | 21946350 | Lung function |
|  | *rs2798641* | 108946847 | Respiratory function tests | NHGRI GWAS catalog | 5.3 | 21946350 | Lung function |
| *SESN1* | *rs2273668* | 109002316 | Glomerular filtration rate | dbGaP | 4.2 | 17903292 | Endocrine function |
|  | *rs2273668* | 109002316 | Creatinine | dbGaP | 4.1 | 17903292 | Endocrine function |
| *PPIL6* | *rs9487094* | 109420812 | Body height | NHGRI GWAS catalog | 5.4 | 18391951 | Growth |
| *FIG4* | *rs9400317* | 109686444 | Hepatitis C | NHGRI GWAS catalog | 5.2 | 19684573 |  |
|  | *rs4947019* | 109768846 | Erythrocyte indices | NHGRI GWAS catalog | 5.1 | 19820697 | Hematopoiesis |
|  | *rs10499054* | 109785031 | Blood pressure | dbGaP | 4.3 | 17903302 | Blood pressure |
|  | *rs4601174* | 109853442 | Heart failure | dbGaP | 3.2 |  | Heart failure |
|  | *rs9374140* | 109929005 | Leukocyte count | dbGaP | 4.6 |  | Hematopoiesis |
| *METTL24* | *rs997865* | 110335462 | Myocardial infarction | dbGaP | 3.7 |  | Heart failure |
| *DDO* | *rs9384741* | 110411533 | Body mass index | dbGaP | 4.7 |  | Growth |
|  | *rs9384741* | 110411533 | Body weight | dbGaP | 4.3 |  | Growth |
| *SLC22A10* | *rs9481083* | 110506679 | Hemoglobin A, glycosylated | dbGaP | 4.0 | 19875614 | Insulin response/growth |
| *SLC16A10* | *rs354524* | 111186906 | Cholesterol, LDL | dbGaP | 4.1 |  | Endocrine function |
| *REV3L* | *rs240993* | 111352511 | Psoriasis | NHGRI GWAS catalog | 19.3 | 20953190 | Inflammation |
|  | *rs7760535* | 111425880 | Metabolism | NHGRI GWAS catalog | 12.0 | 21886157 | Metabolites |
| *TRAF3IP2* | *rs6900341* | 111473206 | Metabolism | NHGRI GWAS catalog | 14.4 | 22286219 | Metabolites |
|  | *rs174376* | 111587162 | Cholesterol | dbGaP | 4.1 | 17903299 | Lipids |
|  | *rs33980500* | 111592059 | Arthritis, psoriatic | NHGRI GWAS catalog | 20.0 | 20953186 | Inflammation |
|  | *rs33980500* | 111592059 | Psoriasis | NHGRI GWAS catalog | 16.0 | 20953188 |  |
| *FYN* | *rs2343601* | 111649496 | Insulin resistance | dbGaP | 4.8 |  | Insulin response/growth |
|  | *rs2343601* | 111649496 | Insulin | dbGaP | 4.1 |  | Insulin response/growth |
|  | *rs9374274* | 111675216 | Insulin resistance | dbGaP | 5.2 |  | Insulin response/growth |
|  | *rs9374274* | 111675216 | Insulin | dbGaP | 4.6 |  | Insulin response/growth |
|  | *rs7746279* | 111678959 | Insulin resistance | dbGaP | 4.9 |  | Insulin response/growth |
|  | *rs7746279* | 111678959 | Insulin | dbGaP | 4.3 |  | Insulin response/growth |
|  | *rs2237259* | 111685346 | Waist-hip ratio | dbGaP | 4.5 |  | Growth |
|  | *rs2237259* | 111685346 | Insulin resistance | dbGaP | 5.3 |  | Insulin response/growth |
|  | *rs2237259* | 111685346 | Insulin | dbGaP | 4.7 |  | Insulin response/growth |
|  | *rs1998038* | 111685792 | Waist-hip ratio | dbGaP | 4.5 |  | Growth |
|  | *rs1998038* | 111685792 | Insulin resistance | dbGaP | 5.3 |  | Insulin response/growth |
|  | *rs1998038* | 111685792 | Insulin | dbGaP | 4.7 |  | Insulin response/growth |
|  | *rs706909* | 111727086 | Triglycerides | dbGaP | 4.1 | 17903299 | Endocrine function |

Table S6 shows results from published genome-wide association studies (GWAS) that include chromosome 6q21. Gene symbol is shown; SNP ID = variant name from the dbSNP database; Location = chromosome position based on GRCh37.p13; Phenotype = major phenotype described in association study with SNP; Data source = reference to association study; *P*-value (–log10) = negative log of the published *P*-value for GWAS; PubMed ID = publication describing the GWAS; Relevant Phenotype = phenotype that may pertain to healthy aging/longevity.

**Suppl. Table S7. Amplification and sequencing primers**

1. ***FOXO3* long-range amplification and sequencing primers**

| Primer name: | Sequence: | Amplicon (bp) |
| --- | --- | --- |
| LR17F | TTTCCAGTCTGTCGCAGGAGCATGTTGCGT |  |
| LR17R | ACTGGCTTCCAAGCACGATGTCGCTGTTCA | 18,872 |
| LR18F | TGCTTGGAAGCCAGTGGGTCATTTGGGTCT |  |
| LR18R | TCAGAAGTTGGCACACTGAGCCCGGGAAA | 18,492 |
| LR19F | TTTCCCGGGCTCAGTGTGCCAACTTCTGA |  |
| LR19R | TCTTCTGTGGCTCCTGGTGCTGCTCATGT | 18,791 |
| LR20F | ACTGAACGAGCACATGAGCAGCACCAGGA |  |
| LR20R | AGGCTGGGTGGTCTTTGCTTCCTGTGGTTT | 18,428 |
| LR21F | AAACCACAGGAAGCAAAGACCACCCAGCCT |  |
| LR21R | ACCCAGCATGCACAGGCATGGACAGAAAGA | 18,428 |
| LR22F | TCTTTCTGTCCATGCCTGTGCATGCTGGGT |  |
| LR22R | TTCTTACAGCTTGGCCTGCACCTGCACCTT | 18,179 |
| LRexon4F | GCAGGCCAAGCTGTAAGAAG |  |
| LRexon4R | TCTCCACTCATTGCCAAATG | 7,419 |

1. **Amplicon 17 Sequencing Primers**

**Forward sequencing primers**

| **Primer name** | **Sequence** |
| --- | --- |
| LR17-1F | ATTAACTCCTCTGCCCCTGC |
| LR17-2F | AGACAATTCCATTTGAAAAGAGC |
| LR17(2)F | TCGGGTCCAAGTTTCAGCTTGTGGGTG |
| LR17-3F | TGAAATTCTCAAGGCTTAATATGCT |
| LR17-4F | AAAATGAAAGAATTAGAAAAGAGGA |
| LR17-5F | AGGATAGCATTTTAACAAAAAGAGGA |
| LR17-6F | TTTCAACACACTGTATTAGGAAAATG |
| LR17-7F | TGAAGAGGCCTTGTCTCACT |
| LR17-8F | GCAGAAGGCAGATAAAGTTCCA |
| LR17-9F | TCCAGTCAGTGCAGTGGTTC |
| LR17-10F | TTTTAAAGCGTGTGTTCATACCA |
| LR17-11F | GCAGTGCCTTGTTACCCAAT |
| LR17-12F | GAGTACTGTGATGTACCACTCTTGG |
| LR17-13F | GAATCTCGCTCTGTCGCCTA |
| LR17-14F | GCAACCAGCCTTCCTTGTAT |
| LR17-15F | AAGAACAACATTATTTCCAATTCAA |
| LR17-16F | AGCCTAGGGATAGAAGAGTGGG |
| LR17-17F | ACACACTTGGTCCTTCAGGC |
| LR17-18F | GCCTGTAATCCCAGCACTTT |
| LR17-19F | TGAGGAGTTTGAGACCAGCC |
| LR17-20F | AATGGCTTGAGAATTCTGTCA |
| LR17-21F | CCATTTTGTTCTGTGTATGTTGG |
| LR17-22F | TCTTCTGATACTGTGATGGTTTCAT |
| LR17-23F | TGGTGTGAGCATGTTTGCTT |
| LR17-24F | AAGTTTTCTCATCAGCCAAAAA |
| LR17-25F | AGGGTTTCCTGGCATGTTCT |
| LR17-26F | TTGGCTTCTAAGGGCAAGAG |
| LR17-27F | AACAAAGCTCTAGCCCCTTGAGT |
| LR17-28F | GCTGGTGGCTTTAGGAATGT |
| LR17-29F | CACGTCAGTTGCTCATCCAT |
| LR17-30F | GTCTCACTCTGTTGCCCAGG |
| LR17-31F | CAAGCATTCACATTGAACACAA |

**Reverse sequencing primers**

| **Primer name** | **Sequence** |
| --- | --- |
| LR17-0R | ACACACTGTCTTGGGGGAAG |
| LR17-1R | CACTGATTAACCCTTCCCATC |
| LR17-2R | TGACACTCAGAAATGGAATTGG |
| LR17(2)R | TGGCTTCCAAGCACGATGTCGCTGTTCAA |
| LR17-3R | TCCCAGGTAATTCAAACATGC |
| LR17-4R | AGTCATCATCCTTCTCCCTGA |
| LR17-5R | TGAGCCAATGTGGAATGATTA |
| LR17-6R | GCCTAAACAGCCTTATCTTTGC |
| LR17-7R | GCAGCAATGACAAATTCACAA |
| LR17-8R | GTCTCAAACTCCTGGCCTCA |
| LR17-9R | GGCAAACAGCACTGAGATGA |
| LR17-10R | ATCCCAAGATCAGGCTTCCT |
| LR17-11R | TGAAAAGTGGAACAAGCGAA |
| LR17-12R | AGGAGAATGGCATGAACCC |
| LR17-13R | CCTCTGCTGGCTCTGAATTT |
| LR17-14R | AGGTGAGAAACCGATATAGCA |
| LR17-15R | TGAATGATTATTAACACTTTGATTTGA |
| LR17-16R | CTGTGAGGACAACCACTCCA |
| LR17-17R | ATGGGGTTTCACCGTGTTAG |
| LR17-18R | AGTAGCTGGGATTACAGGCG |
| LR17-19R | CACTGTGCCTCTGACCCATA |
| LR17-20R | CAGAGTTTCAGTCTATCCTCTGGA |
| LR17-21R | TTGTTTTCAGTGATTTATCAACAGG |
| LR17-22R | GGCTGATCAAGGCCACTAAT |
| LR17-23R | GGGCCCAGAGATAAAAATCC |
| LR17-24R | TCAGAAATGGCTAGACGATCAA |
| LR17-25R | CACATGCATTTATATTAAAGAAGAGGA |
| LR17-26R | GAGGTGAGTATCCTTGCCTTG |
| LR17-27R | CAGGCAACATTCTGAAACAGC |
| LR17-28R | TCCTGATGCCAGATCCTTTC |
| LR17-29R | CAGCTACTTGGGAGCCTGAG |
| LR17-28R | GGCAGTTTGAAGTGTCCCAT |

1. **Amplicon 18 sequencing primers**

**Forward sequencing primers**

| **Primer name** | **Sequence** |
| --- | --- |
| LR18-1F | TGTGTTTTGATAGTGCCTTCCA |
| LR18-2F | AGCAGGCTTGGGCTGAATAG |
| LR18-3F | TTCCCCTGTTCATCTCACCT |
| LR18-4F | AGGAGTGGGCACATAAGCTG |
| LR18-5F | CAGCCATGGTTATTCATCCA |
| LR18-6F | GCCATCTGGAAACTTGCATT |
| LR18-7F | TGACCTCGTGATCCACCC |
| LR18-8F | CCAGTGGCTGTCACTCTGG |
| LR18-9F | TGTTCATCTATCTGGCCTTGTG |
| LR18-10F | CCTCTCAAAGGTGTGAGCCA |
| LR18-11F | ACTCTCCTGCTTTTGCATTG |
| LR18-12F | TGGGCTTGTTCCTTTTGCTA |
| LR18-13F | GGTTTGTTCTCAAGCAACTGG |
| LR18-14F | TGCCATTCCTGACTGTCTTG |
| LR18-15F | TGTATAAAGTTAAATATGAAGACTGGA |
| LR18-16F | GAAAAGAAGTAATGGATAGACAAAAGA |
| LR18-17F | TTACCTGTTAGAACAGAGTTTCCCA |
| LR18-18F | ACAATAAGAAGGAACACGCCA |
| LR18-19F | AGTGTGTGTGGGAAGAGGCT |
| LR18-20F | TTGGTGGGAGAAGGAAAAGT |
| LR18-21F | TTTGGACATCTTTTGGCTTTG |
| LR18-22F | GACCCAGTAGGTGTAGGGCA |
| LR18-23F | TGTAAGAAATGCAACAAAGTTTTCA |
| LR18-24F | ACCTTTCTCTTCCCCATCCT |
| LR18-25F | TTGTTTAATCCATCATATCCCAAA |
| LR18-26F | TGAAATTATGGATTCTGTTGGC |
| LR18-27F | GGAAAATCCCTGTGCATCTT |
| LR18-28F | TTCTCTAATTCTTGTCTGGGCA |

**Reverse sequencing primers**

| **Primer name** | **Sequence** |
| --- | --- |
| LR18-0R | TTCACAGACAGAATGCCTCTC |
| LR18-1R | GGATTCCCAGATGACTTCACA |
| LR18-2R | GTTCCATCATCAATTTGGCA |
| LR18-3R | ATATGGGTTGCCCAGCATTA |
| LR18-4R | CTGCAGACTCACCAGAAGCA |
| LR18-5R | AGCCAAATGAAAATCTAAACCA |
| LR18-6R | GGCAAAGATTCTTCATGGCT |
| LR18-7R | GGTAGAAGCCGACCTTAGGC |
| LR18-8R | CCATACTGCTTCATCTGTAAATCC |
| LR18-9R | CCAACTGAACCAGTCATGGG |
| LR18-10R | CCATCAGGGAAGTCAAGTAAG |
| LR18-11R | TTGATTTTTCATACAGTTTAGCTTTGA |
| LR18-12R | CCCATGCTTACCTAATAAATACTGAA |
| LR18-13R | CACTGGAAACCCGGAAAAG |
| LR18-14R | CCAGCCTGCATGACAGAGTA |
| LR18-15R | CCAAAATTCTGACAAGAACATCA |
| LR18-16R | TTGTGTAACCTAACTGCCCAAA |
| LR18-17R | TCTCCTGTTCAGGCATTTTTC |
| LR18-18R | TTTCAGTATTCGCACAGAAGC |
| LR18-19R | AGCCAGAGCGAGACTCCAT |
| LR18-20R | CACACAGCTTTCTGCCATCT |
| LR18-21R | CATGTTTGGAAAGAGCTGGAG |
| LR18-22R | ATGTAAATGGTCTCAATGAAAGGG |
| LR18-23R | CCTCAAAAACCACTGGGAAA |
| LR18-24R | TGTTCTGCTGATAACTGTTTTCTGTT |
| LR18-25R | TGGAGACTGGTAACGCAATG |
| LR18-26R | TCCATTCCTCCTCTAGGGCT |
| LR18-27R | CATCTCAAGTTTCCTATGTAATAAGTG |

1. **Amplicon 19 sequencing primers**

**Forward sequencing primers**

| **Primer name** | **Sequence** |
| --- | --- |
| LR19-1F | GTGCCAACTTCTGACTGCAA |
| LR19-2F | GGAAAGCTTCTGTGGCTGAC |
| LR19-3F | GCCAGAGATTGCAAAATGAA |
| LR19-4F | CTCTGGGGTTCACTCCATTC |
| LR19-5F | CCCCCTTCTATTGCTGTCTT |
| LR19-6F | TCCCCCTAGTTTTTCTGCTG |
| LR19-7F | AAGGAGGCAAGTGTGCTGTC |
| LR19-8F | TCCCTCACTTTTTGTGTTTTCA |
| LR19-9F | AACAAGTTGAGGATCTCCAGGT |
| LR19-10F | TGAGTGGTTGGAAGGAAAGG |
| LR19-11F | TGAACAAGGAATAATCAGTTTGC |
| LR19-12F | TCACACTCTTTATCATGGGTATCCT |
| LR19-13F | GCTTGCCTCAAATGACACTTC |
| LR19-14F | GAGGTGGGGCAGTATGAGAA |
| LR19-15F | AGCCTTGTGAACAAGCAGGT |
| LR19-16F | TCTTTCATACCTGAAAGGTCGT |
| LR19-17F | AACCCTGTTTTTCTTGCTCTG |
| R19-18F | TCCAGTTCCCTCATTTTATTCA |
| LR19-19F | GCAGAAACCAGGAAACCAAA |
| LR19-20F | CGCCTTAATCCAAATGAACA |
| LR19-21F | TGTTTCTCGCTGCATCTCTC |
| LR19-22F | CCTTCCCCTCCCCATATACA |
| LR19-23F | TAGGACAGGGACTGGGTGAA |
| LR19-24F | TGTAGCCCTTTGTGTTAGCATC |
| LR19-25F | CATTGTAATTTTAATTTGCATTTGCT |
| LR19-26F | TCATCTTTAATTTCTCTCAGCAGTG |
| LR19-27F | AGGTTGCAGTGAGCCAAGAT |
| LR19-28F | GGGATAAATCCCACCTGGTC |
| LR19-29F | TAACTCCCTTTCCCCCTCAT |
| LR19-30F | TGGTTGGAGAATCTGTTTTGTG |
| LR19-31F | TGGATTCCATGCAGATAGCA |
| LR19-32F | CATGTATGTTATAAGCCCCAGAA |
| LR19-33F | GGAGTTTGTTGAGTTTTTCTTGG |
| LR19-34F | ATTCTCCCACCTCAGCCTCT |
| LR19-35F | GTGCTGGGATTACAGGTGTG |
| LR19-36F | AATGCTGCTGTGAACATTTG |
| LR19-37F | TGCAAGGATTTTTATGGGTTTT |

**Reverse sequencing primers**

| **Primer name** | **Sequence** |
| --- | --- |
| LR19-1R | AAGGCTCATATCGATGGAACA |
| LR19-2R | TCATTCCAATCAACCAACCA |
| LR19-3R | ACACAGTGAAACCCCGTCTC |
| LR19-4R | TGAAGAGCAGATTAATGGTTGC |
| LR19-5R | GCTCTCAGACATCAATTCCACTT |
| LR19-6R | TCGGCCTTTATAAAAAGTTATCCA |
| LR19-7R | GCACAACCTCATCGAAGTCA |
| LR19-8R | ATTCCCTCCTCTCCCCATTA |
| LR19-9R | CCATAATTCCAAATTTTAATGACCA |
| LR19-10R | GGCCTGAAACATCCAAACTG |
| LR19-11R | GAGATTGTGCCACTGCACTC |
| LR19-12R | TCCCTGTGCTAACACAAAACA |
| LR19-13R | TTCTGATGAGTCTCCCAGCAT |
| LR19-14R | ACAGGCTGGCAAAAGGACTA |
| LR19-15R | GCTGGGATTACAGGCTTGAG |
| LR19-16R | CATGTCAGCTGGCAGAAAAA |
| LR19-17R | TGAAACAGAGAGAAACTTGAGGTG |
| LR19-18R | CAAAGCCAAAGAAGAGCCTTT |
| LR19-19R | TCCATTTACTCAGCTTACCAGTT |
| LR19-20R | TGGCTAACCCAGAAACACAA |
| LR19-21R | AATCAAGGGCTCTCCAGTGA |
| LR19-22R | ACAAAAGGTGTGGGCGATAG |
| LR19-23R | GAATGAGCCACTGACAAAAGG |
| LR19-24R | GGCAAAAGATTTCAATAGACACG |
| LR19-25R | GCTCTTGGGGATAAATTTCACA |
| LR19-26R | TGAAATAAGGCAAGAAAAAGAAA |
| LR19-27R | TGGTACCAAAAACAGGCAAA |
| LR19-28R | GGGAAAAGTCAGTGGAAGCA |
| LR19-29R | CAAATCACCCAAAACAGCAG |
| LR19-30R | CCCTCCAAATAAAAGCCAGA |
| LR19-31R | AATGAAGAGCGGGGAATGAT |
| LR19-32R | GAAGAGGAAAGAGGGAGAGGA |
| LR19-33R | CTGGGCGACAGAATGAGACT |
| LR19-34R | GGTTGCACAACAGATAGTTGC |
| LR19-35R | CTTGGCAGTTCCTCAAAAGG |
| LR19-36R | CATGCTCATCCTGCTCTTCA |
| LR19-37R | GGTGGTTCTGTGGTGCAGTT |

1. **Amplicon 20 sequencing primers**

**Forward sequencing primers**

| **Primer name** | **Sequence** |
| --- | --- |
| LR20-1F | ATGATGCCAATGCCAAAATC |
| LR20-2F | TGTGGCACCATCTTCTTGAG |
| LR20-3F | CAAGTTGTAGTTTTTAACCTGGGG |
| LR20-4F | TGAGTCTTTCACCATGTTAGTGTTT |
| LR20-5F | GAGGGTCAGGAGAGCGAAG |
| LR20-6F | GTTTCCAGCATCTGGGTTTG |
| LR20-7F | TCGAAATGATTGAGAAATTCGT |
| LR20-8F | AACTGAGTGGAAATGGGTGC |
| LR20-9F | AGATTTGATGGTTAAAAAGATGATGG |
| LR20-10F | GCCAGCCCAGATACCTATGA |
| LR20-11F | GGCTTGGAAATGGGAAGTTA |
| LR20-12F | ATGCATATTTAAGGTACTTAGAGCAGA |
| LR20-13F | ACTCTGCAGCCGGGTTATAG |
| LR20-14F | TTGGAGTTGTGAAGTCAAAGGA |
| LR20-15F | CTTGAGAGGACAGAGAGGGTG |
| LR20-16F | GGTTTTCAGAAGACTGAGGTGG |
| LR20-17F | TGTTGCAGAAACTGGGTCAG |
| LR20-18F | GACCCTTATAGATGTAGAGAAGCCA |
| LR20-19F | TGAGATGTAGAGGCAGTAGTGTTT |
| LR20-20F | AAGGTCACGTGTTTTCAGGC |
| LR20-21F | CATGAAAGAAAAATGCCTGG |
| LR20-22F | GGAATGAGTGGGTAGGGGAT |
| LR20-23F | CCCAGTTACTTGGAAGGCTG |
| LR20-24F | CAGGGGTGGCGAAACGAT |
| LR20-25F | GAAGTGATCCTTGTGCCCAG |

**Reverse sequencing primers**

| **Primer name** | **Sequence** |
| --- | --- |
| LR20-OR | CCCCACGGTTACACTGATTT |
| LR20-1R | CAAATCAAGCTTGGCCCTAT |
| LR20-2R | GTATAGGCTCTGTATCAAATAAAACTT |
| LR20-3R | CTCTCCTATGCACTGCAGGA |
| LR20-4R | CCCCAAAAGGAAATGTCTGA |
| LR20-5R | CTACGGCTCCTGCTGTCTCT |
| LR20-6R | ACAATTCAGATGCAAAATCTTGGT |
| LR20-7R | CTGGGCAACAGAGCAAGACT |
| LR20-8R | TCTCAACACAGCTTCCCAGA |
| LR20-9R | AATGAGGATTGCCTCAAGGT |
| LR20-10R | GACCAGCCTGTATGTGTCCA |
| LR20-11R | GGCTTAGGGACTCTATCTCCAAA |
| LR20-12R | CCACCCAAGATTCTTCCCAT |
| LR20-13R | AAAGCAAAAATATCAGCTAACTAAA |
| LR20-14R | CCACAAAGACATACCTCAGGC |
| LR20-15R | CTTGCTTGCCACTATTTCCC |
| LR20-16R | GTTTCAGGAAATTCAGGCGA |
| LR20-17R | TCTCTCCATATACCCCTCCAAA |
| LR20-18R | TCCAAAGCAGGCTAAGGATT |
| LR20-19R | TCCCCTCAACTTTACCCTCA |
| LR20-20R | ACCTGAACTGCATCTCTTGA |
| LR20-21R | AATAAACCCACCCCCATGTA |
| LR20-22R | GTTGCCCAGGCCACAGTA |
| LR20-23R | CACACAGCCTTACTTACCACCA |
| LR20-24R | TACTGCACAAGCTCACCACC |

1. **Amplicon 21 sequencing primers**

**Forward Sequencing Primers**

| **Primer name** | **Sequence** |
| --- | --- |
| LR21-1F | CCTGTTTTTGTCTTCCTTAATTGTC |
| LR21-2F | AGTGCATCCTCTCCTCATGG |
| LR21-3F | GGTGTGGAGGAGTCATTTTCA |
| LR21-4F | AGCATCGCATTGTGGAATTT |
| LR21-5F | CTCCCACTGGGTGAGAGAAG |
| LR21-6F | CTTACGCACCTTCCTTTTCT |
| LR21-7F | TTCATTGGATAAGAAAGGTAGGGA |
| LR21-8F | GAGGTTTTTGATACTTCACAATTTCA |
| LR21-9F | CCTGACCTCAAGCGATCTG |
| LR21-10F | TATGTCTTTGCGTTCCCCTG |
| LR21-11F | TTTTGAGAGCAGTACCCAGG |
| LR21-12F | TTTGATACTCAAGGAAAAGGTAAAGA |
| LR21-13F | TTAGTTCATTCTTGTAATTTTACTGTG |
| LR21-14F | GTGTTTGCCCCTCAGATCAT |
| LR21-15F | TGATACTGCTGTTGCCAAGG |
| LR21-16F | TGTAATTATGTATGTATTTGTTTGCTT |
| LR21-17F | TTCCCAGTAAATGGTAAGACC |
| LR21-18F | GGGTAGGTATAGAATATAGGCACA |
| LR21-19F | ATCCGAATGGAGGCCAAA |
| LR21-20F | TCAAAGGTTGTGTGCAGTATCA |
| LR21-21F | TCTTGGAGATGGGAAACCTG |
| LR21-22F | AAAGGACAGAAAAACCACGG |
| LR21-23F | CCCTAATTTACTCCTGGATGCC |
| LR21-24F | TGTGGGAGAGCTAAGTGGAAA |
| LR21-25F | GGAGAAGAGAAGAAAGATGGCT |
| LR21-26F | AGATAGGCCCAGGTTGATGG |
| LR21-27F | GCTAACATTCCACATAATGCCTC |
| LR21-28F | GCACCCAGTTATAGGAATTTT |
| LR21-29F | TGCAAAACCACTATGGTGAAA |
| LR21-30F | TTCATGTGAAGGAAGGGATTG |
| LR21-31F | GGATATGTAGAAGTTCAGTTTATGGC |
| LR21-32F | GAGGAGACTTTAGAACCAGAATGTG |
| LR21-33F | AGTGTGCTAATCCAGACATTGG |
| LR21-34F | TTGGAGAAAGACAACAGGGG |
| LR21-35F | TTGCTGCTTGAAAACTTAAATG |

**Reverse sequencing primers**

| **Primer name** | **Sequence** |
| --- | --- |
| LR21-OR | GATTCACATGTTCACAAGGAGC |
| LR21-1R | AACTACAGGGGAAGAAAACCAA |
| LR21-2R | AAGCTCTGGTTAAAGGGGGA |
| LR21-3R | TGTGTTGGGTCTTATTGCTCC |
| LR21-4R | CCAAAGAGGTCCAGGAAAGA |
| LR21-5R | TTGATCATTCAAGTTTTGTCTTCTT |
| LR21-6R | TCCAAGACTGTCCCTAAGGC |
| LR21-7R | CCCAACATAAAAGGGCAAGA |
| LR21-8R | TTTAAAGGCATAGTAAGAAAATCAAAA |
| LR21-9R | TTGTGGCAATTCTAGCCCTC |
| LR21-10R | AGTGAAACCCTGTCTCAAAAA |
| LR21-11R | TCCTGTCGTTTGCTAGCCTT |
| LR21-12R | TGCCTCCAGCCAGTTTTAAT |
| LR21-13R | TTTGATAAGTTCCCACTTGCTTC |
| LR21-14R | GGAGAAAGGAGTCACCACTGA |
| LR21-15R | TGGTCCCTGGTCTCATTCAT |
| LR21-16R | GCAATTCTGGGGATTCAAGA |
| LR21-17R | TGGTAATTAGAGGTTTCTGTTCATGT |
| LR21-18R | TGCAAGACTATTTAAAGGCAGC |
| LR21-19R | TGGATTGGTTGGTGAAAGTG |
| LR21-20R | AATGCAGGAGGAAAGAAGCA |
| LR21-21R | TGGGAAGAGTTAGATAGCAAAAGA |
| LR21-22R | TCTCCCTACATCAATCAGAAAA |
| LR21-23R | CAGAGGTGCTTCCAGGTCTC |
| LR21-24R | TCCACTGCTTTAGCTTGTGAAC |
| LR21-25R | GAGCAAAGGGCAGACATACTT |
| LR21-26R | TGAGCCATTTTCAGGTTCAA |
| LR21-27R | ATCTATCACCCAAAGGGCAG |
| LR21-28R | TGAAATTCAGCTCTAATGAGGA |
| LR21-29R | TGGGTGAGTTAAACAGCTTGAG |
| LR21-30R | CAGACAGACCTCAAAGGGACA |
| LR21-31R | GCCCCCATTACTTGGTAATTC |
| LR21-32R | AGCAGAGACAGACTCACACCA |
| LR21-33R | GGAAGAAGCATCTAAAGGCG |
| LR21-34R | CAGTTAGAAACAAGATTTCCCG |

**(g) Amplicon 22 sequencing primers**

| **Primer name** | **Sequence** |
| --- | --- |
| LR22-1F | GAATTGCCTTTGAAAGATCCTG |
| LR22-2F | TGTTGCTTCCAGACAAAAACC |
| LR22-3F | TAATTGCCAGATTCTGCTAGGTG |
| LR22-4F | GGAAGGCACATCCCTAGTTC |
| LR22-5F | TCCCCTGCGTATAACAAGGA |
| LR22-6F | ATTACAGGTGTGAGCCACCG |
| LR22-7F | TTCCTATTCTTTCTTAAATGTGAAATG |
| LR22-8F | GGGGCATTTTGCAGTTCTAT |
| LR22-9F | TCCTGAACTACAGGCCCTTC |
| LR22-10F | AAATTAGCCAGGTGTGGTGG |
| LR22-11F | GCAGTTGTTGCAGACAAATTAGA |
| LR22-12F | TCAGCTCATCTTATCCTTGTAGA |
| LR22-13F | CCAGGAATTTGAGGAAAACT |
| LR22-14F | CTTTTCTCCCTGCAGAACTCC |
| LR22-15F | GCCCACTGGGGGACTCAT |
| LR22-16F | ATGGGCCATGAGAAGTTCC |
| LR22-17F | AGAGAAGGAGAGGGATGGGA |
| LR22-18F | TGGGTGGTAAAGCAAGAACC |
| LR22-19F | TCCCAGGCTGGAGTACAGTG |
| LR22-20F | AACCCTCTGCCATGATGAAG |
| LR22-21F | GGATTGTACAGAAAATTAAGGGAGA |
| LR22-22F | TGTGATGCTGAATCTGAGCC |
| LR22-23F | TCAAAAGAAGACAGCCTCATCA |
| LR22-24F | GCAGGCTGCAGTATTGGAAG |
| LR22-25F | TCCTGGAAAGCAGTGACATTT |
| LR22-26F | AGATTCTCCACCTTGCAGGA |
| LR22-27F | TGCTGAAAGGAGTCAGCACC |
| LR22-28F | CAGAGGTGCATGTGGTGTG |
| LR22-29F | GTCCTGCAGTCAAGTCCCTC |
| LR22-30F | GGTGTTGAGGAGAGCTGTTTG |
| LR22-31F | TTACTGTGATACAATTTGAGGTTCA |
| LR22-32F | TGAAGGTTTGGATGTCAGTACG |
| LR22-33F | TAAGTAATCAGAAAGTGTCTTGACTG |

**Reverse sequencing primers**

| **Primer name** | **Sequence** |
| --- | --- |
| LR22-0R | AGAGGTCTGCGTGCCACA |
| LR22-1R | CTTCAGCCCTTCCAAACCAT |
| LR22-2R | ATTTCACAGTCCTGCTGCCT |
| LR22-3R | GCTTGACAGAACGAGAATGATTT |
| LR22-4R | GCCCAGTTACCCCTCAAAAT |
| LR22-5R | AGTGGCCATGATCACATTCA |
| LR22-6R | CCAAATTATGCAGAAGTCACCA |
| LR22-7R | CCCTTAAAAAGCATTTAGGGAA |
| LR22-8R | CCCAGCCCAGAATGTTTTA |
| LR22-9R | CTCACTGCAACCTCCGCCT |
| LR22-10R | CCAGTTGACAGTGATGACCC |
| LR22-11R | GGGGGAAAAGAGCAACTTTA |
| LR22-12R | TGGCAGTTTTCATATCTAGGGG |
| LR22-13R | TCTTGCCAGTTCCCTCATTC |
| LR22-14R | TGTTAAAGGAGCTGGTTGGG |
| LR22-15R | CATGAGTTCACTACGGATAATGGA |
| LR22-16R | ATTGACAAAAGCGGTGACG |
| LR22-17R | TTTGGAAACTGCTGATATCATTTA |
| LR22-18R | CGCCTGTAGTCCCAGCTACT |
| LR22-19R | AACATTCCCCTTTCTGCAAC |
| LR22-20R | TGCAGTCATTTATTATCATCACCA |
| LR22-21R | AGCCCGCCAGAGAAGCAT |
| LR22-22R | AGTGCTGGGATTACAAGCGT |
| LR22-23R | TCAAACATCAGTCAAACAAGCA |
| LR22-24R | GCCAGAGGTGAAAACAGGTG |
| LR22-25R | GAGGATTGGGGAAAGCAATA |
| LR22-27R | TTTTTGTGGAATAGACCATGTGA |
| LR22-28R | TCTAGGAGCTCTGTGAGGCA |
| LR22-29R | CCAGACAGCTGGGACTTCTC |
| LR22-30R | TTTTTCTGGGTTTTCACTCAA |
| LR22-31R | GGGAGACGGAGAGAAAGAAGA |
| LR22-32R | TTTGTTACTCGGTGGGCAG |

**(h) Amplicon sequencing primers for exon 4**

**Forward sequencing primers**

| **Primer name** | **Sequence** |
| --- | --- |
| LRex4-1F | TTACACCTGCATTTCCACCC |
| LRex4-2F | GGTTCTGCCTGGACTCAGAC |
| LEex4-3F | TCTTTGAACCTGACCCATCG |
| LRex4-4F | TCGCTAAATTTAAGCAACAGAAAA |
| LRex4-5F | GGGGCAAACTTTCCTCAAAT |
| LRex4-6F | GAGAGAGGGCGTCTTCCCTTA |
| LRex4-7F | CCCAAGCTAAGTATCATTGACTTTC |
| LRex4-8F | CTTCACAGGTGACCCCAAC |
| LRex4-9F | AAGTGTTATTGTGAGGGGAAAAA |
| LRex4-10F | AAAGGTGGGAGAAGCAGATG |
| LRex4-11F | GCAACATAGCAAGACCCCAT |
| LRex4-12F | CATGGAAAGTCCCATGTCCT |
| LRex4-13F | TTGTACAGGGTGGTGAGCC |
| LRex4-14F | CAGTAGGGCCTGTGATTTCC |
| LRex4-15F | TGGGACCACCTTTGGTACAT |
| LRex4-16F | GTCTGCTTGTCAGTGAGCCA |
| LRex4-17F | GCAGCACAAAACAGATCAGG |
| LRex4-18F | CAGGGCTCCAGAACCTGATA |
| LRex4-19F | AAAATTAGCCTGGCATGGTG |
| LRex4-20F | TGTGTCTGGTCACTTATTTCTCTAAAA |

**Reverse sequencing primers**

| **Primer name** | **Sequence** |
| --- | --- |
| LRex4-0R | TGGGGTGTGTGTATGTGGG |
| LRex4-1R | TCTTCCTCTTTGCCAACTCAA |
| LRex4-2R | GGTTCCACTGTCCTCATCCA |
| LRex4-3R | GCCATCCTCATACTTCATAGCTG |
| LRex4-4R | GTTGAAACCTTCACATTCACAAA |
| LRex4-5R | AGGCAATATGCCAATTAACAA |
| LRex4-6R | AGGACAGAAAGAGTAGAGGGAGC |
| LRex4-7R | ATCAAAAGGCCCCAATCTTC |
| LRex4-8R | ACCTTGCCCTGCAAAGTATG |
| LRex4-9R | ATCACATTATATACATCTGCTTCTCCC |
| LRex4-10R | AGATCGCACCACTGCACTC |
| LRex4-11R | TGTTTTGGTGGGTCCTTTTT |
| LRex4-12R | AACACCACAGAATGGCCG |
| LRex4-14R | ATGCAAAGAAAAGAGTCTTCATA |
| LRex4-15R | CAGCACATTACTAAAGAATCCAGG |
| LRex4-16R | GCTGCACTTGACTTTACCCTG |
| LRex4-17R | GTACACTGAGCCTGGCAGC |
| LRex4-18R | ATCTCGGCTCACTGCGAC |
| LRex4-19R | CATAATGTGCTAGGACAAGTATACAA |

Table S7 shows: (a) *FOXO3* long-range amplification and sequencing primers. PCR was used to generate 7 chromosome 6-specific products of 16–18 kb in length in order to sequence the entire *FOXO3* gene. These amplified products were then used for sequencing by capillary electrophoresis using the individual primers shown in parts (b)–(h) of the Table.
